# What is known about the quality of out-of-hospital emergency medical services in the Arabian Gulf States? A systematic review

**DOI:** 10.1371/journal.pone.0226230

**Published:** 2019-12-19

**Authors:** H. N. Moafa, S. M. J. van Kuijk, G. H. L. M. Franssen, M. E. Moukhyer, H. R. Haak

**Affiliations:** 1 Faculty of Public Health and Tropical Medicine, Jazan University, Jazan, Saudi Arabia; 2 CAPHRI Care and Public Health Research Institute, Maastricht University, Maastricht, The Netherlands; 3 Department of Clinical Epidemiology and Medical Technology Assessment, Maastricht University Medical Centre+, Maastricht, The Netherlands; 4 Maastricht University Library, Maastricht, The Netherlands; 5 Faculty of Applied Medical Sciences, Jazan University, Jazan, Saudi Arabia; 6 Department of Internal Medicine, Maxima Medisch Centre, Eindhoven, The Netherlands; 7 Division of General Internal Medicine, Department of Internal Medicine, Maastricht University Medical Centre+, Maastricht, The Netherlands; Radboud University Medical Center, NETHERLANDS

## Abstract

**Background:**

The Emergency Medical Services (EMS) have been developed in the Arabian Gulf States (AGS) in the last three decades. The EMS needs continuous quality assessment of their performance to improve and provide the best out-of-hospital care. This study aims to assess the quality of EMS in the AGS according to the six quality domains of the Institute of Medicine.

**Methods:**

We searched four databases (i.e., PubMed, EMBASE, Web of Science, and CINAHL) for studies that reported on the quality of EMS in any of the AGS using clinical or non-clinical performance indicators. To quantify study quality and risk of bias, the adapted Newcastle Ottawa Scale was used. We focused on structural and functional indicators, clinical and non-clinical.

**Results:**

Twenty-five studies were eligible for inclusion. One study contained result of safety, fifteen time-centeredness, twenty effectiveness, five patient-centeredness, and thirteen studies reported on equity of EMS. None of the studies reported on efficiency of EMS. A significant proportion of studies showed high scores on the Newcastle-Ottawa scale. Limited studies on EMS quality were available, not covering all relevant quality domains and not covering the whole AGS region. The equity domain showed the best outcome performance finding, whereas finding of the patient-centeredness domain showed room for improvement in the foreseeable future.

**Conclusion:**

This review highlights the need for more and better studies of sufficient quality about all domains of quality in EMS in all the AGS. EMS research in Kuwait and Bahrain is warranted, as currently studies of EMS quality are unavailable for these States. Moreover, efficiency researches exploring this discipline should be conducted specially no studies were found has been searching this domain.

**Trial registration:**

PROSPERO registration number: CRD42019123896.

## Introduction

The Arabian Gulf States (AGS) are composed of six nations (i.e., Saudi Arabia [SA], Oman, the United Arab Emirates [UAE], Qatar, Kuwait and Bahrain). These countries provide Emergency Medical Services (EMS) for all different levels of emergency in all regions. They have full collaboration with all governmental and non-governmental health sectors e.g. Primary Health Centers and Hospitals, and also with non-health sectors such as Civil Defense, Polices, and Municipalities. In Addition, they have many types of ambulance vehicles equipped with prescribed standards such as defibrillators, ventilators, and cardiac resuscitation medications. They triage cases based on a specific triaging system guideline to identify the emergency severity and urgency level. They then dispatch the appropriate vehicle(s), through utilizing a Computer Aided Dispatch System (CAD), to the scene from one of many scattered ambulance stations. All EMS providers in AGS can treat patients on the scene without transporting them once their clinical condition does not urge the necessary transportation. Additional characteristics of EMS stratified by country are shown in S1 Table. The EMS in SA is provided at no direct cost to the general public by the Saudi Red Crescent Authority (SRCA), and has played a critical role in assuring the local public that medical aid is readily available and just a phone call away regardless of the distance.[1]

The EMS in the AGS, like that of any nation, needs continuous quality assessment of their performance indicators, so that they can provide the best possible out-of-hospital care. In 2001, the National Academy of Medicine (NAM) formerly known as Institute of Medicine (IOM), identified 6 quality domains for improving health care systems: safety, time-centeredness, effectiveness, patient-centeredness, efficiency, and equity.[2, 3] The World Health Organization (WHO) highly recommends these domains for national health systems and policy makers to reform and optimize outcomes.[4]

In some other parts of the world, institutions provide guidelines and recommendations to aid in obtaining the highest quality of EMS. For example, the Joint Royal College Ambulance Liaison Committee (JRCALC) in the United Kingdom provides guidance to general practitioners, Out-of-Hospital care providers, ambulance technicians, and paramedics. Its guidelines provide valuable information and treatment algorithms to follow in case of emergency events, such as acute coronary syndromes (ACS), asthma, abdominal pain, trauma and many others.[5] The US has The American Ambulance Association (AAA),[6] and the National Association of State EMS Officials (NASEMSO),[7] both of which play a pivotal role in promoting high-quality care for acutely ill and injured clients, improving the quality and efficiency of state EMS program administration. No such organizations are available for the AGS, nor has much research been performed to quantify and compare performance indicators for the current state of the EMS in the AGS on the whole.

The aim of this study is to assess the quality of EMS systems in the AGS according to the six quality domains of the IOM. To do so, we performed a systematic literature review and identified quality indicators of the six predefined quality domains.

## Materials and methods

This review was reported in accordance with the Preferred Reporting Items for Systematic Reviews and Meta-Analyses (PRISMA) statement, see S2 Table, PRISMA 2009 checklist.[8] A protocol for this review was registered with PROSPERO, an international database of prospectively registered systematic reviews. (record CRD42019123896; CRD = Centre for reviews and Dissemination).

### Search strategy

We considered studies eligible for inclusion if they reported on the quality of EMS in any of the AGS using performance indicators. Studies were excluded if they were not written in English or Arabic, they solely investigated the quality of intra-hospital emergency healthcare, or if they focused on intra-hospital patient’s transportation. The search was carried out in four databases (i.e., PubMed, EMBASE, Web of Science, and CINAHL) from inception up to June 1^st^, 2019. In each database, a sensitive search was performed using the following search term domains: emergency medical services, quality, and region. A fully reproducible search can be found in S3 Table.

### Study selection

**Fig 1:**
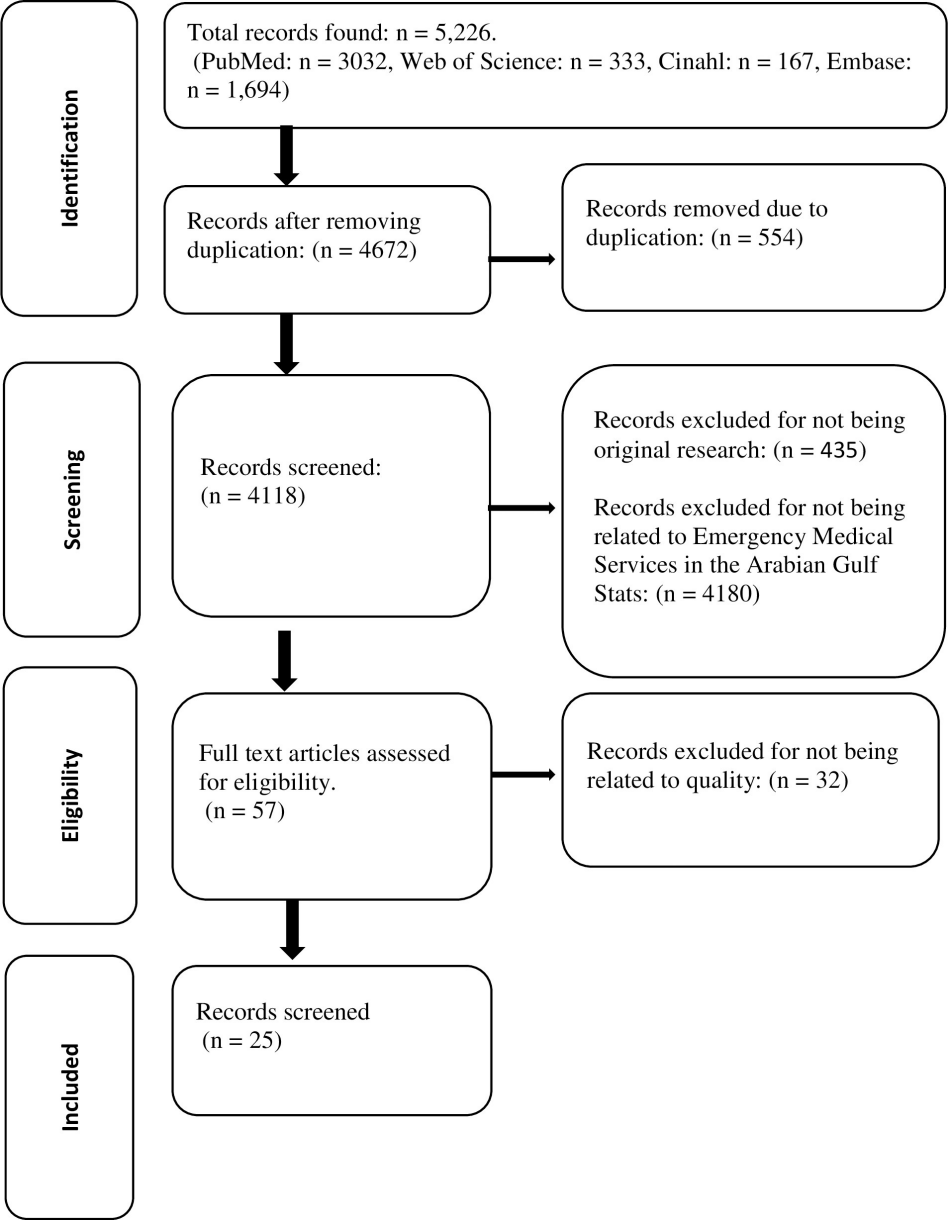
Flow chart of study selection process.

Two authors (HM and SvK) independently screened for potentially eligible articles based on titles, abstracts and, if necessary, full text. Additionally, the reference sections of all selected manuscripts were screened for studies that might have been missed with the search strategy. In case of disagreement, consensus was reached by discussing the study in the study group during a face to face meeting. Fig 1 shows the search results per search engine and the process of exclusion of irrelevant articles.

### Data extraction

All relevant data of the studies eligible for inclusion were extracted, i.e.: authors, date of publication, country or countries in which the study was performed, study design, particulars of the EMS, process and outcome measures that were relevant for any of the following domains of quality of care: safety, time-centeredness, effectiveness, patient-centeredness, efficiency and equity. To quantify study quality and risk of bias for all of the evaluated studies, the Newcastle Ottawa scale was used after it was adapted by Herzog et al.[9] The adapted Newcastle Ottawa scale consists of items to measure the quality of cross-sectional and cohort studies.

### Data synthesis

Meta-analysis has not been considered due to the heterogeneity of study objectives, and design.

## Results

The initial search identified 5226 references. In total, 554 duplicated records were removed. After applying the exclusion criteria in the primary screening by title and abstract, we removed another 4,615 records. Next, we read the remaining fifty-seven articles. As a result of the secondary screening, we excluded thirty-two studies because they were not related to the quality of out-of-hospital emergency medical services in the AGS. Finally, twenty-five studies were included for this systematic review. Nineteen out of these twenty-five studies were conducted in one of four different States: eight in SA, four in UAE, five in Qatar, and two in Oman. In addition, two studies were conducted in multiple Asian countries, and included data from the UAE-Dubai ambulance system. The other studies provided data of multiple Gulf States together. Studies from Kuwait and Bahrein were not available. The included studies are summarized in Table 1.

**Table 1:** Characteristics and data extraction of the included studies.

No	Author and year of publication	Study location	Study Design	Goal & Objective	Quality Domains	Sample N
1	Abdelrahman, H., et al. (2014) [10]	Qatar	Cross-sectional, retrospective analysis of all trauma-related deaths between January 2010 and December 2012	• to analyze the time-based trauma mortality	• Time-centered Effectiveness • Equity	333 clients
2	Al-Ghamdi, A. S. (2002). [11]	Saudi Arabia	Cross-sectional study. Data collected between October 1999 and January 2000	• to analyze the EMS response times	• Time-centered	874 emergency calls
3	Al-Shaqsi, S., et al. (2014). [12]	Oman	Cross-sectional, retrospective study of road traffic trauma clients admitted to the Sultan Qaboos University Hospital between January and December 2011	• to assess the differences in the outcome of road traffic trauma clients between those transported by EMS and those privately transported.	• Effectiveness • Equity	821 EMS clients and 273 non-EMS clients
4	Al-Thani, H., et al. (2014). [13]	Qatar	Cross-sectional, retrospective study on medical records of all trauma clients who required intubation between January 2010 and December 2011	• to analyze the outcome of PHI versus ERI.	• Effectiveness	482 clients over 2 groups based on location of intubation procedure (PHI: 239, ERI: 243)
5	Alanazi, A. F. (2012). [14]	Saudi Arabia	Cross-sectional questionnaire-based study was undertaken among paramedics in 2011	• to investigate the barriers faced by EMS providers in the city of Riyadh	• Patient-centered	140 paramedics
6	AlHabib, K. F., et al. (2014). [15]	Multiple Arabian Gulf States	Prospective multicenter, cross-sectional study (Gulf RACE-2 study) among clients with a diagnosis of STEMI or unstable angina and non-STEMI recruited between October 2008 and June 2009 at 65 hospitals.	• to determine the rate and predictors of EMS use and to compare the clinical presentation, management, and short- and long-term outcomes for clients with ACS who used EMS versus those who did not.	• Time-centered Effectiveness • Equity	5,184 clients
7	AlHabib, K. F., et al. (2016). [16]	Multiple Arabian Gulf States	Cross-sectional, retrospective analysis of data of a prospective study (Gulf RACE-3study) among consecutive clients with acute STEMI that were treated in hospitals in SA, Oman, UAE, Kuwait, Qatar, and Bahrain from 1 January 2014 to 15 January.	• to assess temporal changes in clinical arrivals and EMS usage rates • to describe the EMS system • to compare clinical presentation, management, and outcomes in the ED and in the hospital between clients that used EMS and those that used other means of transport, and between hospitals that provide PCI versus those that do not.	• Time-centered • Effectiveness • Equity	2,928 clients
8	AlHabib, K. F., et al. (2019).[17]	Saudi Arabia	Prospective, multi-center, study included all consecutive hospital admissions of patients with AMI, Between May 2015 and January 2017	• to evaluate the clinical characteristics, management, and outcomes of a representative sample of patients with acute myocardial infarction (AMI) in Saudi Arabia.	• Effectiveness • Equity.	2233 clients
9	Alrazeeni, D. M., et al. (2016). [18]	Saudi Arabia	Cross-sectional, retrospective analysis of patient care reports of non-transported emergency calls documented by 10 EMS stations in Riyadh, SA, between March and May 2014	• to determine the epidemiology of non- transported EMS calls • to identify factors that contribute to non-transport of clients • to recommend suggestions for reduction in number of non-transported calls	• Time-centered • Patient-centered • Equity	1,791 clients
10	Althubaity, E., et al. (2013). [19]	Saudi Arabia	Cross-sectional face-to-face interviews using a structured questionnaire at 17 EMS centers between October and December 2011	• to assess the knowledge, experience, and the impact of seniority of Saudi EMS personnel in dealing with acute stroke clients	• Effectiveness	120 paramedics
11	Batt, A. M., et al. (2016). [20]	United Arab Emirates	Prospective cohort study of all presenting cases of OHCA between February 2014 and March 2015 in the Northern Emirates service area.	• to report the characteristics of OHCA clients and their outcomes	• Time-centered • Effectiveness • Equity	384 clients
12	Bin Salleeh, H. M., et al. (2015). [21]	Saudi Arabia	Prospective cohort study of all presenting adult cases of OHCA between July 2012 and September 2013 at KKUH, ED, Riyadh.	• to report the characteristics and outcomes of OHCA clients	• Effectiveness Equity	96 clients
13	Callachan, E. L., et al. (2016). [22]	United Arab Emirates	Survey of 195 physicians present at the hospital ED and were involved in care of clients with suspected ACS in Abu Dhabi between June 2012 and December 2013.	• to describe the perceptions towards EMS among physicians caring for clients with STEMI for 1) The likelihood of advising an ACS patient to use EMS to go to the hospital, 2) Satisfaction with the current EMS level of care given to ACS clients, 3) Likelihood of using the EMS for themselves or their family if a cardiac emergency occurs, and 4) Opinions regarding the steps that they felt could be taken to further improve EMS and Out-of-Hospital ACS care.	• Patient-centered	106 physicians
14	Callachan, E. L., et al. (2016). [23]	United Arab Emirates	Semi structured interviews during an 18-month, multicenter, prospective study of consecutive clients admitted with STEMI to four government hospitals in Abu Dhabi.	• to estimate utilization, knowledge, and perceptions of EMS among clients with STEMI	Time-centered Effectiveness Patient-centered	587 clients
15	Callachan, E. L., et al. (2017). [24]	United Arab Emirates	Retrospective review of EMS and hospital data obtained through chart review supplemented with prospectively collected follow-up data over a period of 18 months, with follow-up interviews at 30 days, six months, and one year after initial discharge.	• to assess differences in demographics, medical history, treatment times, and follow-up status among clients with STEMI who were transported to the hospital by EMS or by private vehicle, or were transferred from other medical facilities.	• Time-centered • Effectiveness	455 clients
16	Dhaffar, K. O., et al. (2005). [25]	Saudi Arabia	Cross-sectional evaluation of SRCA evaluation forms filled out by the doctor on duty in the ER from 06 November 2002 to 5 December in Makkah.	• to evaluate the clients and the general appearance of the team and their cooperation	• Effectiveness	632 clients
17	El-Menyar, Ayman., et al (2018) [26]	Qatar	A retrospective observational analysis was conducted on all patients with TBI who were admitted directly to the Hamad Trauma Center (HTC), from January 1, 2010 to December 31, 2014.	• to report the predictors and temporal patterns of death in moderate-to-severe isolated and polytrauma brain injuries in relation to their admission to the only national level 1 trauma center and comparing survivor to non-survivor.	• Time-centered	810 clients
18	Fares, S., et al. (2011). [27]	Multiple Arabian Gulf States	Prospective, multicenter, study of consecutive clients hospitalized with ACS in 65 centers in five AGS (Kuwait, Oman, UAE, Yemen, Qatar, and Bahrain). Clients were enrolled in a pilot phase that lasted for 1 month in May 2006, and a subsequent study phase from January 2007 to June 2007.	• to examine EMS use by clients with ACS	• Time-centered • Effectiveness • Equity	7,859 clients
19	Hamam, A. F., et al. (2015). [28]	Saudi Arabia	Cross-sectional observational study through interviewing the general public in public venues during 01 July 2010–31 Dec 2010. The survey consisted of a two parts questionnaire. The first part was completed for all subjects. The later was completed only for those subjects that had previous experience with the SRCA service in Jeddah.	• to investigate the level of public awareness of the EMS system	• Time-centered • Patient-centered	1,534 participants from the general public
20	Irfan, F. B., et al. (2016). [29]	Qatar	Cross-sectional, observational study with prospective enrollment of OHCA clients from 1st June 2012 to 31st May 2013. Data were collected from incident reporting and dispatch data, EMS Out-of-Hospital care records, and patient medical records from 4 EDs and 8 hospitals. Follow-up was through access of hospital medical records and was censored at the date of death or up to 3 years from enrollment. Data for the OHCA registry were collected on all OHCA clients resuscitated by EMS	• to describes the epidemiology, EMS, and outcomes of OHCA.	• Time-centered • Effectiveness • Equity	447 clients
21	Irfan, F. B., et al. (2017). [30]	Qatar	Retrospective, cross sectional, observational study that analyzed data from the HTC, trauma registry and OHCA registry. Data were collected on OHCA after trauma of clients from 1 January 2010–31 December 2015.	• to measure the outcomes of OHCA after trauma who determined by EMS and having received Out-of-Hospital cardiopulmonary resuscitation. • to determine the predictors of survival.	• Time-centered • Effectiveness • Equity	410 clients with OHCA after trauma
22	Nadar, Sunil K., et al. (2018) [31]	Oman	This retrospective study that took place between January 2012 and December 2016 at the Sultan Qaboos University Hospital (SQUH), Muscat, Oman. All adult clients who presented following an OHCA to the Emergency Department of SQUH during the study period were included.	• to describe the epidemiological patterns of patients presenting to a tertiary care centre in Oman following an OHCA event. In addition, to assess the survival rate and demographic and angiographic features of resuscitated OHCA patients.	• Effectiveness	216 clients
23	Ong, Marcus Eng Hock., et al. (2015) [32]	Multiple Asian Countries	prospective, international, multi-center cohort study of OHCA in the Asia-Pacific. Twelve sites from the seven PAROS countries participated in the study. The 12 sites were UAE (Dubai) is part of them. Each site contributed 1–3 years of data during the study period. data from January 2009 to December 2012.	• describe the characteristics of OHCA transported by EMS and outcomes across the network sites.	• Time-centered. • Effectiveness • Equity	66395 clients, of which 405 from Dubai, UAE
24	Shehab, A., et al. (2014)[33]	Multiple Arabian Gulf States	Prospective multicenter, cross-sectional study (Gulf RACE-2 study) among clients with a diagnosis of STEMI who admitted for reperfusion therapy and received pPCI and recruited between October 2008 and June 2009 at 65 hospitals.	• to explore the quality of pPCI practice and its impact on morbidity and mortality.	• Time-centered • Effectiveness	3432 clients
25	Tham, L. P., et al. (2018). [34]	Multiple Asian Countries	Retrospective, cross-sectional analysis of PAROS study data from January 2009 to December 2012. PAROS is a prospective, observational, multi-center cohort study in the participating PAROS sites (12 sites from seven countries). The 12 sites were UAE (Dubai) is part of them. Each site contributed 1–3 years of data during the study period. data from January 2009 to December 2012.	• to describe the characteristics and outcomes, and to find factors associated with survival after pediatric OHCA.	• Effectiveness • Equity	974 clients, of which 17 from the UAE

ACLS, Advanced Cardiac Life Supports; AMI, Acute Myocardial Infarction; CPR, Cardio Pulmonary Resuscitation; CVS, Cardiovascular System; DNT, Door to Needle Time, EMS, Emergency Medical Services; ED, Emergency Department; ER, Emergency Room; ERI, Emergency Room Intubation; HTC, Hamad Trauma Centre; IQR, Interquartile Range; IV, Intra Venous; KKUH, King Khalid University Hospital; MEDEVAC, Medical Evacuations; OHCA, Out-of-Hospital-Cardiac-Arrest; PAROS, Pan-Asian Resuscitation Outcomes; PCR, Patient Care Report; PHI, Pre-Hospital Intubation; ROSC, Resuming of Spontaneous Circulation; SA, Saudi Arabia; SRCA, Saudi Red Crescent Authority; STEMI, S-T Elevated Myocardial Infarction; t-PA, Tissue Plasminogen Activator; UAE, Unites Arab Emirates

Table 2 shows the results of applying the Newcastle-Ottawa scale to the included studies. Ten studies were scored on a six-point scale because multiple items were not relevant for those specific studies. The remaining fifteen studies were scored on an eight-point scale. The item pertaining to the Ascertainment of Exposure was not applicable to any of the included studies, so it was omitted from the scale entirely. The item on comparability between the respondents and non-respondents was applicable to only fourteen studies of which eleven failed to report anything concerning that item. Most studies scored well on the following items of the Newcastle-Ottawa scale: representatives of the sample, sample size, and the use of a validated measurement tool. Moreover, the reliance of many studies on data registries is associated with a low probability of bias while the reliance of few studies on self-reporting might introduce self-selection bias.

**Table 2:** The result of the critical appraisal for the included studies based on the adapted Newcastle-Ottawa scale.

Selection						Comparability Max 2*	Outcomes		
No	Study (First Author)	Representativeness of the sample	Sample Size (max 1*)	Non-respondent	Ascertainment of Exposure (max 2*)		Assessment (max 2*)	Statistical Test- confidence interval	Total score
1	Abdelrahman, H., et al. (2014) [10]	registry, selection bias unclear	*	probability of missing data high	NA	NA	**	*	5 out of 6
2	Al-Ghamdi, A. S. (2002). [11]	*	*	*	NA	NA	**	Not used	5 out of 6
3	Al-Shaqsi, S., et al. (2014). [12]	*	*	*	NA	** EMS group compared to non-EMS	**	*	8 out of 8
4	Al-Thani, H., et al. (2014). [13]	*	*	*	NA	** group intubated by EMS compared to group intubated by ER	**	*	8 out of 8
5	Alanazi, A. F. (2012). [14]	*	*	did not show total number, missing data unclear	NA	NA	*	Not used	3 out of 6
6	AlHabib, K. F., et al. (2014). [15]	registry, selection bias unclear	*	*	NA	** compares groups	**	*	7 out of 8
7	AlHabib, K. F., et al. (2016). [16]	registry, selection bias unclear	*	*	NA	**compares groups	**	*	7 out of 8
8	AlHabib, K. F., et al. (2019).[17]	registry, selection bias unclear	*	*	NA	** compares groups	**	*	7 out of 8
9	Alrazeeni, D. M., et al. (2016). [18]	*	*	*	NA	NA	**	Not used	5 out of 6
10	Althubaity, E., et al. (2013). [19]	*	*	*	NA	** compares 3 groups		*	6 out of 8
11	Batt, A. M., et al. (2016). [20]	*	*	*	NA	NA	**	*	6 out of 6
12	Bin Salleeh, H. M., et al. (2015). [21]	single center	small sample	*	NA	** Traumatic OHCA compared to non-traumatic OHCA	**	*	6 out of 8
13	Callachan, E. L., et al. (2016). [22]	*	*	did not show total number, missing data unclear	NA	NA	**	*	5 out of 6
14	Callachan, E. L., et al. (2016). [23]	*	*	*	NA	** EMS group compared toh non-EMS	**	*	8 out of 8
15	Callachan, E. L., et al. (2017). [24]	selection bias likely	*	did not show total number, missing data unclear	NA	**	**	*	6 out of 8
16	Dhaffar, K. O., et al. (2005). [25]	*	*	probability of missing data high	NA	NA	**	Not used	4 out of6
17	El-Menyar, Ayman., et al (2018) [26]	registry, selection bias unclear	*	*	NA	**compares 2 group	**	*	7 out of 8
18	Fares, S., et al. (2011). [27]	registry, selection bias unclear	*	*	NA	** EMS group compared to non-EMS	**	*	8 out of 8
19	Hamam, A. F., et al. (2015). [28]	*	*	did not show total number, missing data unclear	NA	NA	*	Not used	3 out of 6
20	Irfan, F. B., et al. (2016). [29]	*	*	*	NA	** compares 2 groups	**	*	8 out of 8
21	Irfan, F. B., et al. (2017). [30]	*	*	*	NA	** compares 2 groups	**	*	8 out of 8
22	Nadar, Sunil K., et al. (2018) [31]	single center	small sample	*	NA	**compares 2 groups	**	*	6 out of 8
23	Ong, Marcus Eng Hock, et al. (2015) [32]	single center	*	did not show total number, missing data unclear	NA	NA	**	*	4 out of 6
24	Shehab, A, et al. (2014) [33]	registry, selection bias unclear	*	*	NA	** compares 2 groups	**	*	7 out of 8
25	Tham, L. P., et al. (2018). [34]	single center	small sample	did not show total number, missing data unclear	NA	NA	**	*	3 out of 6

*value equal to one for scoring, EMS: Emergency Medical Services; OHCA: out-of-Hospital cardiac arrest; NA; not applicable

All the included studies were evaluated according to the six quality domains defined by the NAM (safety, time-centeredness, effectiveness, patient-centeredness, efficiency, and equity). A brief summary of the most relevant results stratified by these quality domains is shown in Table 3.

**Table 3:** Summary of findings related to the six different quality domains.

Quality Domain	Finding	Sources
**Safety:** Avoiding harm to clients from the care that is intended to help them.[5]	1. In Riyadh, SA, a face-to-face cross-sectional interview with EMS provider has shown that around 98% of participants dispatched their clients to the nearest hospitals without considering the availability of stroke treatment facilities.[19]	[19]
**Time-centeredness** Reducing waits and sometimes harmful delays for both those who receive and those who give care.[5]	1. A study in Gulf States (excluding SA, but including the non-gulf state of Yemen) compared the usage of private transportation and EMS for ACS clients. The study found that among EMS users, reperfusion therapy for STEMI/LBBB clients was significantly less likely (20% vs. 31%: P < 0.001). [27] 2. A study in multiple Gulf States (excluding SA, but including Yemen, which is not a gulf state member) compared the demographic and clinical characteristics of STEMI clients who received timely primary percutaneous intervention (pPCI). They reported door to balloon time (D2B) ≤ 90 minutes versus delayed pPCI (D2B > 90 minutes). Compared to being transported by private means, an ambulance ride was associated with shorter D2B, but this was not statistically significant (30 of 45 [66.7%] patients transported by ambulance had a D2B ≤ 90 minutes, 78 of 153 [51.0%] transported privately, P = .063).[33] 3. Study conducted in 5 out of 6 Gulf States (except Kuwait) found that clients with STEMI, who arrived by EMS, had a significantly longer interval from symptom onset to hospital arrival (median 184, range 111–370 vs 173, 90–358 minutes; P = .018), but a similar percentage of clients presented to the ED within 12 hours of symptom onset. They also had similar rates of receiving thrombolytic therapy and shorter door-to-needle time (DNT; median 35, range 23–60 vs 40, 25–65 minutes, P = .01) but were less likely to receive primary PCI (26.7 vs 35.5%, P = .04. Furthermore, he found that There was no significant difference in median length of stay for those who transport by EMS compare with Private Transport P = 0.284. [15] 4. Another Study conducted in 5 of 6 Gulf States (except Kuwait) found that the median (IQR) from symptom to ED time was significantly shorter for Out-of-Hospital EMS 144 (IQR 191) when compared to inter-hospitals ambulance transportation 230 (IQR 277), and private transportation 185(IQR 241), P< 0.001.[16] 5. In SA, a study evaluating the EMS rescue times found that the mean response time was 10.23 min (S.D = 5.66 min). The average EMS time was 61.19 min (SD = 16.86 min) and the average scene time was 15.2 min. In addition, 85% of the incidents took up to 66 min or less to be completed. [11] 6. Another study in SA found that the response time was within 25 minutes in 80.9% of calls; 65% of calls were responded to within the first 15 minutes. The response time was limited to half an hour in 82.9% of calls. The average response time was lower at locations near the dispatching EMS centers: sometimes as low as 5 minutes. [18] 7. In Jeddah, in SA, study found that according to people perception. The majority estimated that the estimated time arrival ETA of an ambulance response to their home to be about 30 minutes or more. [28] 8. In UAE, EMS transportation was associated with a shorter time to treatment in the Hospitals when compared with other modes of transportation in Abu Dhabi.[24] 9. In UAE, the median EMS response time was 9 minutes (IQR: 6 to 14). In 75% of cardiac arrests, the EMS response time was 14 minutes or less.[20] 10. Another study performed in UAE found a median EMS response time of 9 minutes (IQR 6 to10), and a median scene time of 9 minutes, (IQR 4–13).[34] 11. Study described EMS performance toward OHCA in UAE, Dubai, found the EMS response time was 10 minutes (IQR 7 to 12).[32] 12. A study in Qatar found a median EMS time for trauma clients who died (6.8% of all) of 60.5 Minutes (range: 3–160).[10] 13. Another study in Qatar described the presentation, and outcome of traumatic brain injury (TBI) for two groups of clients who survived and those who died. It showed the scene time is significantly higher for non-survival in compare with survival [17] minutes (IQR 1 to 90), Vs 23 minutes (IQR 1 to 110), P value = 0.009]. The median of total EMS time for both of group was 60 Minutes (IQR 3 to 234) without any significant difference for both groups. [26] 14. The EMS response time was less than eight minutes for 25% of transported case in Qatar, while 75% took more than 8 minutes. On average, scène time 26.5 minutes (SD 12.1) was higher than response time 14.6 Minutes (SD 8.7), and transportation time 22.7 Minutes (SD 12.7). [13] 15. In Qatar, the median response time was 8.72 min (IQR 6.8 to11.8). The median scene time was 37.9 min (IQR 28.0 to 50.6), and the median transport time was 21.4 min (IQR 13.7 to 31.5).[29]	[27] [33] [15] [16] [11] [18] [28] [24] [20] [34] [32] [10] [26] [13] [29]
**Effectiveness:** Providing services based on scientific knowledge to all who could benefit and refraining from providing services to those not likely to benefit (avoiding underuse and misuse, respectively).[5]	1. Study in Gulf states include Yemen, non-gulf state, and exclude SA measured the utilization of ACS clients for EMS compare with Private transportation. The study found 17% of ACS clients used EMS. Regarding the Crude rates of in-hospital outcomes by mode of presentation to the ED, it was found Clients transported by EMS had higher rates of in-hospital mortality, cardio- genic shock, and stroke (p < 0.01 but when confounding factor adjusted for age, gender, and presentation characteristics, the associations between EMS utilization and in-hospital outcomes were no longer statistically significant. [27] 2. A study in multiple Gulf States compared two groups of clients. The first group of clients received timely pPCI (D2B ≤ 90 minutes) and were compared to delayed pPCI (D2B > 90 minutes). The study showed that the use of ambulance services was substantially low (<30%) in both groups[33] 3. Study conducted in 5 of 6 Gulf States, except Kuwait, found that 25% of ACS clients use EMS for the transportation to high facility hospital. They observed a higher CVS complication, and mortality rate in the EMS group, but this could be explained by confounders such as age, risk factors (OR 1.54, 1.12–2.13) and at 30 days (OR 1.37, 1.04–1.8) and 1 year (OR 1.41, 1.12–1.78) after hospital discharge, but that difference became non-significant after multiple adjustments. [15] 4. Other study in gulf state without Kuwait found 3.7% of STEMI clients were transported by Out-of-Hospital EMS while 22% were transported from non- PCI hospital to PCI hospital. Worthwhile, three fourth of STEMI clients were transported to PCI and non- PCI hospitals by private.[16] 5. A study in SA in a small sample of clients: (n = 96) showed that two third of OHCA after trauma were transported by EMS while the rest were transported by private transport. Of all non-traumatic OHCA clients, only one third were transported by EMS, the rest by other means. Meanwhile, none of the cases who were transported by EMS had Resuming Of Spontaneous Circulation ROSC before arrival to ED. [21] 6. Another multicenter study in SA described the total admission of acute myocardial infarction. The study showed that 5.2% (n = 96) transported by Saudi EMS to Emergency department in 50 hospitals of 13 provinces in SA. [17] 7. An interview with SA EMS providers showed that 78% had no knowledge of stroke subtypes, and most of them had previous more than or equal to five years experiences. Meanwhile, only 10% of those EMS providers with five or more year of experiences were aware of tPA. 94% of all participants were unaware of Tissue Plasminogen Activator t-PA. [19] 8. In Makkah, during the seasonal Muslim gathering in one month, one study in SA observed that 2.5% of ER attendee were transported by ambulance and the vast majority of clients transported by EMS whom require airway management (80%), and breathing support (92.4%) did not received it [25] 9. In Riyadh, the capital city of SA, a retrospective study to identify factors contribute to EMS non-conveyance of clients reported that the non-conveyance rate was 25% while only 3.9% of non-conveyance’s cases treated at the scene [18] 10. In UAE, study found that 60% of Clients with STEMI transported by private and 12% by EMS while 28% was inter-hospital clients’ transportation. Those STEMI clients that were transported by EMS did not receive ECG during transportation. The study also observed a higher mortality rate in the EMS group, but this could be explained by confounders such as age, risk factors, and socioeconomically status.[24] 11. In Abu Dhabi, UAE, study measures the STEMI clients perception, utilization, and knowledge toward EMS which found 15% of clients had transported by EMS while 85% transported by non-EMS vehicle. [23] 12. Prospective study to describe EMS performance toward OHCA in the Northern Province of UAE, found that 99.5% of all OHCA cases were transported by EMS to hospitals and EMS provide shock rhythm analysis for 17% of transported clients. In addition, 71% of cases received mechanical chest compression devices, 84% of clients received laryngeal mask airway management, and ROSC was resumed for 3.1% of OHCA by EMS. [20] 13. Study to describe EMS performance toward pediatrics OHCA of UAE, found that all clients were transported by EMS. However, EMS providers used the automated external defibrillator for 11% of child with OHCA; inotropic medications were given for 6% of clients by EMS. In addition, EMS gave Intravenous Dextrose for 75% of clients. ROSC was resumed in 6% of clients by EMS, and 6% of clients were received laryngeal mask airway management by EMS. [34] 14. Study to describe the UAE-Dubai EMS performance toward all transported OHCA clients, found that (n = 46) 11.4% of transported clients have been witness by EMS providers. 13% (n = 6) of them has ROSC.[32] 15. Study to describe the Qatar EMS performance toward all transported OHCA clients due to cardiac reasons, found that 80% of clients are non-shockable and EMS provide shock rhythm for the other 20% shockable clients. 95% of EMS clients received ACLS and mechanical chest compression devices for 70% of EMS Clients. ROSC was resumed for 13% of OHCA by EMS. [29] 16. In Qatar, study to describe the outcomes of OHCA after trauma found that 98% of cases transported by EMS. EMS defibrillated 10.2% and three fourth of clients received ACLS and control bleeding was done for 10%. [30] 17. In Qatar, EMS transported 94% of acute severe traumatic clients who required intubation, while 6% were transported by private vehicle. Of those transported by EMS, 45% were intubated during transportation. PHI was associated with high mortality when compared with ERI. However, selection bias could not be ruled out and therefore, PHI needs further critical assessment. [13] 18. In Qatar, 91.3% of trauma-related death cases had been transported by ambulance, and 4.8% were transported by private car. [10] 19. In Oman, private vehicles transported 33% of all trauma clients while 67% were transported by EMS. Those traumatized patents who transported by EMS had a statistically non-significant 36% reduction in mortality compared with privately transported clients. Analysis showed no significant difference in short- and long-term outcomes for both group of clients. The EMS-transported group had a lower mortality rate compared to the non-EMS group (5.3% vs 8.1%; p = 0.67). [12] 20. Another study in Oman measuring epidemiology and outcome of OHCA clients who admitted to single tertiary hospital found that EMS transported 1.4% (n = 3) of total clients arrived to hospital whereas 98.6% (n = 213) arrived by private.[31]	[27] [33] [15] [16] [21] [17] [19] [25] [18] [24] [23] [20] [34] [32] [29] [30] [13] [10] [12] [31]
**Patient-centered:** Providing care that is respectful of and responsive to individual clients preferences, needs, and values and ensuring that clients values guide all clinical decisions.[5]	1. In Jeddah, in SA, study found that 33% of people did not know the call number; 94% said that MEDEVAC is needed. Furthermore, 17.7% of people still found it unacceptable for male paramedics to respond to a female emergency unescorted by a male family member. The client’s preference rate to request EMS for their relatives with cardiac arrest was 57%. It also shows that (70%) of client were satisfied about the services had been given to them. [28] 2. In a survey among EMS providers in three major cites of SA, it was found that 60% of EMT stated that the presence of family and bystanders, and the impression of people and family, were the most two agreed upon barriers for the participant. The third barrier was traffic congestion with 54.8%. Although, over half reported that clients did not resist their treatment, 60% of them reported they think clients have an unfavorable impression of EMS providers. [14] 3. In Riyadh, the capital city of SA, a retrospective study to identify factors contribute to EMS non-conveyance of clients, the study shows that 54% of the client refused to be transported via themselves and their relatives. [18] 4. In Abu Dhabi, UAE, study shows that Less than half of the physicians were "Somewhat Satisfied" (35%) or "Very Satisfied" (7%) with current EMS level of care for S-T Elevation Myocardial Infarction STEMI clients. Most participants were "Very Likely" (67%) to advise a clients with a cardiac emergency to use EMS, but only (39%) felt the same for themselves or their family in Acute Coronary Syndrome ACS in Abu Dhabi, UAE. [22] 5. In Abu Dhabi, UAE, study found around 55% of participants stated that the EMS telephone number is unknown to them. It is worthwhile to note, around half of clients prefer private because it is quicker than EMS; 13.4% stated that private transport is easier to access; 8% of clients stated that they select private because they thought that their symptoms were not cardiac related. [23]	[28] [14] [18] [22] [23]
**Efficiency:** Avoiding waste, including waste of equipment, supplies, ideas, and energy.[5]	No study found measuring this domain.	
**Equity:** Providing care that does not vary in quality because of personal characteristics such as gender, ethnicity, geographic location, and socioeconomic status.[5]	1. Study conducted in 5 of 6 Gulf States, except Kuwait, found that 81% of ACS clients who were transported by EMS were male and more than half were Gulf State citizens. [15] 2. Study conducted in 5 of 6 Gulf States, except Kuwait, found that 17.5% of Gulf citizens used EMS during STEMI attacks. Furthermore, clients earning up to 5000 per month were more likely to take the EMS in case of STEMI compared to clients earning over $5000 per month (odds ratio [OR]: 1.6). [15] 3. In Riyadh, SA, a study to identify factors contributing to EMS non-conveyance of clients reported that the male to female ratio for non-transported cases was 2:1 where males account for 51.9%, and female’s clients account for 23.4%. Meanwhile, in a significant number of cases (24.7%) the gender was not listed in the clients care reports (PCR). [18] 4. In Makkah the rate of male to female percentage transported by EMS: 67.8%. Vs 32.2%. [25] 5. Study in SA described the total admission of STAMI clients to 50 Saudi’s hospitals. The study showed that 5.2% (n = 61) of STAMI clients who admitted to emergency department were transported by Saudi EMS. Male were 86.9% (n = 53) and female 13.1% (n = 8). [17] 6. Study in UAE; found that individuals from the Indian subcontinent represented the largest group of OHCA, accounting for 38.8% of all cases, while clients from other Arab countries represented 23.7% of all cases. The UAE nationals accounted for 16.7% of cases. Male percentage were 76% while females were 24% for all transported OHCA clients. [20] 7. Study to describe EMS performance toward pediatric OHCA in UAE, found that 76.5% of pediatric out of hospital cardiac arrests who transported by EMS were male. [34] 8. Study described the EMS performance toward OHCA in UAE-Dubai found that 82.7% (n = 335) of transported client were male sex. [32] 9. Study in Qatar analyzing the time-based mortality trauma clients shows the male: 95% (n = 316) female: 5% (n = 17).[10] 10. Study in Qatar comparing the successful intubation rate in field, and in ER, it shows the male (95%) female (5%).[13] 11. Study in Qatar found 92% of Out of Hospital Cardiac Arrest clients after trauma who were transported by EMS were male while 7% were Female. 25% were Middle Eastern, 37.6% South Asian, 4% African, and in 28%, ethnicity was not mentioned.[29] 12. Study in Qatar showed that the majority of cases were male (80.5%) with a median age of 51 years (IQR = 39–66). Frequently observed ethnicities of OHCA clients were Qatari (19.9%) and South Asians (45%); Indian (16.6%), Nepalese (11.6%), and Pakistani (6%). [30] 13. In Oman, study of show that both ethnicities Omani and non-Omani were transported. It is worthwhile to note, the significance of more male clients was represented in the EMS compared with the non-EMS group (72.8 vs 63.4, p value 0.006). [12]	[15] [16] [18] [25] [17] [20] [34] [32] [10] [13] [29] [30] [12]

ACLS, Advanced Cardiac Life Supports; CPR, Cardio Pulmonary Resuscitation; CVS, Cardiovascular System; D2B, Door to Balloon time; DNT, Door to Needle Time, EMS, Emergency Medical Services; ED, Emergency Room; ERI, Emergency Room Intubation; IV, Intra Venous; MEDEVAC, Medical Evacuations; OHCA, Out-of-Hospital-Cardiac-Arrest; pPCI, primary percutaneous intervention; PCR, Clients Care Report; PHI, Pre-Hospital Intubation.; ROSC, Resuming of Spontaneous Circulation; STEMI, S-T Elevated Myocardial Infarction; SA, Saudi Arabia; t-PA, Tissue Plasminogen Activator; UAE, United Arab of Emirates.

### Safety

Only one study reported results that were relevant for the Safety domain.[19] This study from SA showed that 98% of EMS providers who participated dispatched clients who were having a stroke to the nearest hospital without considering the availability of stoke treatment facilities (i.e. that have radiological and therapeutic interventional ability).

### Time-centeredness

Seven studies reported response times specifically for life-threating cases with emergency level one such as Out-of-Hospital Cardiac Arrest (OHCA), ACS, and stroke, and two measured all emergency cases’ response times. [18–29,12,24,32,34] In addition, three studies measured the total EMS time. Response time is defined as the period of time that begins with EMS activation (i.e., a phone call to the dispatcher) and ends with the arrival at the location of the patient. Total EMS time is the response time plus the time to deliver the patient to the hospital. The median response time for OHCA varied between 8.7 min in Qatar and 9 min in the UAE.[20,29] In Qatar, the median total EMS time was 60.5 min (range: 3 to 160) for trauma clients who died on the scene or during transportation to the ER.[10] One study found the response time in Riyadh, SA to be 10.23 min on average.[11] Another SA study reported that 81% of calls were responded to within twenty-five minutes or less and 65% were responded to in fifteen minutes or less.[18] A Saudi study conducted in Jeddah, found the majority of civilians estimated that the response time of an ambulance to their home would be about thirty minutes or more in SA.[28]

The time to get access to hospital facilities was evaluated after clients had been delivered to the ER by EMS. One study conducted in the UAE found EMS transportation was associated with a shorter time to treatment in the hospitals when compared with other modes of transportation.[24] A study conducted in multiple countries of AGS to compare the usage of private transportation Vs EMS for clients with ACS found that among EMS transported clients, reperfusion therapy was significantly less likely for ST-elevated myocardial infarctions (STEMI) and for left bundle branch block (LBBB) clients compared to other modes of transport due to the major delay from the onset of clients’ symptoms to ER arrival (20% vs. 31%: P<0.001).[27] A study in multiple Gulf States (excluding SA, but including Yemen, which is not a gulf state member) compared the demographic and clinical characteristics of STEMI clients who received timely primary percutaneous intervention (pPCI). They reported door to balloon time (D2B) ≤ 90 minutes versus delayed pPCI (D2B > 90 minutes). Compared to being transported by private means, an ambulance ride was associated with shorter D2B, but this was not statistically significant (30 of 45 [66.7%] patients transported by ambulance had a D2B ≤ 90 minutes, 78 of 153 [51.0%] transported privately, P = .063). [33] Another study conducted in multiple AGS found that clients with STEMI, who arrived by EMS, had a significantly longer median interval from symptom onset to hospital arrival compared to clients transported by private means (184 vs 173 minutes; P = 0.018). However, a similar percentage of transported clients arrived at the ER within 12 hours of symptom onset in both groups. They also showed similar rates of receiving thrombolytic therapy but the median door-to-needle time was shorter for EMS-transported clients (35 vs 40 minutes, P = 0.01) However, they were less likely to receive primary percutaneous intervention (PCI) (26.7 vs 35.5%, P = 0.04). Furthermore, the authors found no significant difference in median length of stay for those who transport by EMS compared with private transport.[15] The last study found that the median time from symptom to ER was significantly shorter (144 minutes) for Out-of-Hospital EMS time when compared to private transportation (185 minutes), (P<0.001).[16]

Information about time management indicators stratified by urgency classification and triage was lacking in all of the included studies for this review.

### Effectiveness

Twenty studies considered effectiveness of EMS.[10–29,18,24–32] The studies concentrated on topics such as the percentage of clients in life-threating situations that are taken to the hospital via EMS, interventional therapy provided by EMS providers during transportation, mortality and morbidity for EMS clients during transportation or after delivery to ER staff, and the outcomes of EMS clients such as resuming of spontaneous circulation (ROSC). In addition, results were presented on emergency medical technician (EMT) qualifications.

Regarding transportation rates, 17 to 25% of all ACS clients were transported by EMS in the AGS.[15,27] The overall transportation rate irrespective of the nature of the emergency was only 3.7% by Out-of-Hospital EMS, 22% inter-hospital EMS transportation, and 75% by private transport.[16] A study in multiple Gulf States compared two groups of clients. The first group of clients received timely pPCI (D2B ≤ 90 minutes) and were compared to delayed pPCI (D2B > 90 minutes). The study showed that the use of ambulance services was substantially low (<30%) in both groups.[33] Dhaffar et al. found that 2.5% of emergency room ER clients were transported by EMS to ER, while 97.5% came to ER by other means.[25] A study in Oman found that 33% of trauma clients were transported by EMS.[12] The percentage of clients with OHCA of cardiac origin that were transported by EMS was about 1.4% in Muscat, Oman, 33% in Riyadh, SA, 99.5% in UAE, and 100% in Qatar. For ACS clients it was 12% in the UAE, and 15% specifically in Abu Dhabi, UAE whereas 5.2% in SA.[17–21,20,24,29] In the UAE, all pediatrics OHCA clients were transported by EMS.[34] The percentage of transported cases of OHCA after trauma was 66% in Riyadh, SA and 98% in Qatar.[21]^,^[30] Furthermore, in Qatar, 91.3% of deceased clients had been transported by EMS.[10]

With regard to interventional procedures during transportation, one study found that not in all participating AGS Electro-Cardio-Grams (ECGs) were performed during EMS transportation.[16] In the UAE, no ECGs were performed for any patient during transportation.[24] In the SA, 94% of all EMS providers were unaware of the value of tissue plasminogen activator (t-PA) for stroke cases.[19] In Qatar, a study showed that 55% of clients in urgent need of endotracheal intubation were not intubated during transport.[13] Four studies conducted in Qatar and UAE showed partial protocol adherence; exemplified by the fact that not all of OHCA clients in these two countries received advanced cardiac life support (ACLS).[20,29,30,34]

Concerning the morbidity and mortality of clients who utilized the EMS, two studies used data of the Gulf State Registry, and three studies were conducted in UAE, Oman and in Qatar. They found that the mortality and morbidity are higher for the clients who were transported by EMS compared to private transport, but were unable to sufficiently control for confounding variables.[12,15,24,27]

Finally, five studies measured the ROSC rate of EMS clients before ER arrival. The result varied considerable from 0 to 13% (SA [adult] (0%), UAE [adults] (3.1%), UAE [pediatrics] (6%), Qatar [adult] (13%), and UAE-Dubai [adult] (13%).[20,21,29,32,34]

### Patient-centeredness

Five studies discussed the patient-centeredness of EMS in AGS.[14,18,22,23,28] Two studies found that 33% and 55% of people did not know the emergency telephone number in SA and the UAE respectively.[23]^,^[28] Regarding the client impression and satisfaction, Alrazeeni et al. showed that 54% of clients refused transportation of themselves or their relatives in SA.[18] Another study in SA showed that 70% of clients were satisfied about the services that had been given to them.[28] Furthermore, about 60% of paramedics from three major cities in SA reported that they felt that unfavorable impressions that people have of EMS affects their performance. A small percentage of the respondents (17.7%) reported the need for female paramedics to respond to female clients whereas 94% stated that ambulance by air was urgently needed.[14] While the other study related to the impression that civilians have of EMS in the UAE found that around half of clients did not prefer EMS transportation due to its slowness; others preferred private transportation because of easy accesses (13.4%).[23] In addition, a study in the UAE found that less than half of doctors working in a hospitals rated their satisfaction for EMS provider as ‘Somewhat satisfied’ (35%), or ‘very satisfied’ (7%). However, most participants were ‘very likely’ (67%) to advise a patient with a cardiac emergency to use EMS, but only (39%) felt the same for themselves or their family in case of ACS.[22]

### Equity

Thirteen studies reported on equity.[10–29,15,18,21–25,27,30–32,34] Males were far more prominent in demanding EMS services.[10–29,15,18,21–25,27,30,34] Socioeconomic status and its relation to citizenship also had a significant role in requesting EMS services. One study conducted in multiple AGS States found that only 17.5% of Gulf citizens used EMS during STEMI attacks, compared to 82.5% of non-citizens staying in the same States.

Furthermore, clients earning up to $5000 per month were more likely to take the EMS in case of STEMI compared to clients earning over $5000 per month (odds ratio [OR]: 1.6).[16] Two studies conducted in Qatar and one in the UAE showed that most non-Qatari (80%) and non-Emirati (83.3%) ethnicities utilized EMS transportation more than the Qatari and Emirati citizens.[20,29,30]

### Efficiency

None of the included studies reported on characteristics of EMS relevant for this domain.

## Discussion

In this systematic literature review we described the current status of the quality of EMS in the AGS. To the best of our knowledge, we are the first to do so. However, no studies included in this review described EMS quality for Kuwait or Bahrain. This could be due to their small geographical size and demographics. Even though one study from Kuwait was recently published that described the hospital’s response toward victims who had been exposed to a mosque bombing and showed that 67% of clients had been transported into emergency department of Kuwait hospital in less than 22 minutes, but this study did not provide information about the mode of transportation (EMS versus private vehicle). Therefore the study was excluded from analysis. [35]

Our review identified one study that looked at EMS safety.[19] This study shows inappropriate hospital transportation for stroke clients in SA. The Saudi Arabian Health System classifies hospitals into primary (public), secondary, and tertiary hospital according to the available capacity in terms of equipment, specific specialty, and availability of trained staff. For example, public hospitals in SA do not have technical equipment for the procedures of cardiac catheterization. Hence, clients who have ischemic heart attack would not benefit from the transportation to primary hospitals. Only secondary and tertiary hospitals have the capability of treating stroke clients because of availability of advanced radiology and fibrinolytic medication such as t-PA. Therefore, the different categories of hospitals require the SA EMS policy makers to educate their providers about different emergency cases continuously. They also need to help them to illustrate which hospitals are capable of treating so that the clients are not transferred to inappropriate facilities.

Other important safety findings, such as adverse event rate, wrong medication rate, patient fall rate, and incident report rate, were not available for any of the AGS. A scoping review of Fisher et al. illustrated that the cornerstone of safety assessment is the culture of reporting.[36] Hence, culture of reporting should be established in the AGS EMS systems, making it easier to assess safety in the future. Research should be developed with consideration to adverse event reporting, and should introduce clinical audits.

The total EMS time is a highly important factor for life-threatening diseases. We did not find any study in the AGS that measured any performance indicators of on-phone triaging time. In addition, no studies were found pertaining to search the urgency type that consider measuring the response time based on emergency cases level; starting from life threating up to non-emergency cases. We found the researchers in AGS did not consider the “golden hour” to be a benchmark during their measurement of EMS time. They measured different EMS time intervals and the total EMS time. The median response time in UAE and Qatar was comparable to the response times observed in the US. In the US, for example, a multicenter study found that the median response time for clients calling from either urban or suburban areas was about six minutes. For rural areas, the response time was almost double with a median of thirteen minutes.[37] However, the studies included in this review did not describe the EMS time in rural regions. It is worth mentioning that we did not find any studies measuring EMS intervals in SA focusing only on life-threating cases. We argue that response time should be documented by objective quantitative tools through reviewing information report or using information system technology.

Variation of time spent on the scene is subject to many different confounding factors such as severity of the case, geography, and type of EMS system protocol. Therefore, prolonged scene time cannot be considered as a negative indicator per se, unless the client’s outcome such as his medical condition and satisfaction were measured and show a negative outcome due to waste of effective time in non-indicated procedures. Irfan et al. found that in Qatar the median scene time for highly critical cases was more than half an hour.[29] This finding could be due to the fact that in Qatar ACLS has only recently been introduced and might be still under further strategic development.[38,39] Another study in Qatar showed that case severity and scene time affect total EMS time.[10] Demography and cultural factors may also affect the scene time in AGS. For example, female clients often need their own relative to be beside them during paramedic examinations. Another example is mass gathering of people at the scene during road traffic accidents.[40] Hence, sometimes the increased scene time is a result of community culture rather than of slowness of the system. Therefore, in order to identify the effectiveness of prolonged scene time, further research is still needed in the AGS.

None of the included studies considered the time of delivery of clients to ER staff. Undoubtedly, Hospitals resource levels and type (private or governmental), and ER bed consumption have a major role in this specific time. We found only one study that showed the time of delivery of clients but did not point to the type of emergency cases being delivered nor to the category of hospital [11].

Future research for EMS time domain could identify obstacles related to time management and it could provide solutions that improve outcomes. In general, the times to delivery to ER staff are important, irrespective of the possible underlying disease. The researchers in AGS should avoid benchmarking the EMS total time with the “golden hour” when they are planning to measure the time without stratifying on severity of cases or urgency type. In addition, high-quality studies are still needed, particularly, studies that focus on time attributes, such as call activation to first defibrillation time, crews’ scene arrival to first defibrillation time, time from collapse to first ROSC, and from arrival to initial CPR performed.

Studies that reported on the effectiveness domain showed the impact of process attributes on outcome finding. For example, the conveyance rates of OHCA or non-life-threating cases and compliance and adherence to EMS protocol or guidelines were often accompanied by outcome finding such as ROSC, survival rate to ER, or mortality rate. We found that all but one study showed a high conveyance rate of OHCA.[10,20,21,30,34] Bin Salleeh et al. found an alarmingly low conveyance rate of OHCA clients to hospital.[21] Moreover, they found ROSC and survival rates of zero percent, likely a consequence of the low transportation rate. In contrast, the reasons given for low ROSC rate in Qatar and UAE were partial adherence and compliance to protocol. The ROSC rate in Qatar and UAE were high in comparison to SA, but lower when compared to the US.[41]

The study by Bin Salleeh et al was not the only study showing low EMS utilization.[21] Dhaffar et al. reported very low conveyance rate of EC to Hospital in Makkah, and the partial adherence to protocols as well.[25] The low OHCA conveyance rate particularly in SA, the partial adherence to protocols in many AGS, and the low ROSC rate should be considered major challenges. A recent SA review concluded that socioeconomic status could be an important factor for the willingness of people to use private cars of transportation instead of EMS to reach the ER.[40] A US study showed that despite that 85% had insurance coverage that included eligibility to be transported by ambulance, 78.4% of them showed willingness to seek ER treatment by any alternative means, while 61.6% of the total were willing to come by private car.[42] Another study in the US showed that 88.7% of participants would first call EMS call number, if one of them or their relative will have recognized multiple symptoms of stroke.[43] Those studies showed that further research on those who choose EMS and those who choose using private transportation in AGS to identify this performance procedure are necessary.

A study conducted in Qatar identified the success rate of interventional endotracheal intubation. It showed that endotracheal intubation by the EMS crew was performed on only half of clients.[13] This could be lack of competency of EMS providers or could be due to large number of clients that were difficult to intubate. The incompetency of adherence to protocols is not only a challenge for AGS. Ebben et al. found many different internal and external factors affecting the EMS providers adherence to protocol.[44] Consequently, the AGS stakeholder should overcome those obstacles affecting their EMS providers of completely adhere to the national protocols.

The non-conveyance rate in an urban area of SA was shown to be low, mainly because of refusal of transportation.[18] Unfortunately, the study did not provide reasons for refusal. Reasons could be related to clients’ satisfaction, but further research is needed to explore non-conveyance reasons. A recent Dutch study showed very similar non-conveyance rates in the Netherlands, and explored numerous reasons for non-conveyance.[45]

Accessing EMS by phone is free of charge in AGS. Two studies identified moderate rates of unawareness of EMS phone numbers in UAE and SA.[23,28] The finding of unawareness of EMS number intersect with other SA studies in this review conclude the low EMS utilization.[15–25,21,40] Moreover, the two states have a massive influx of foreigners because of trading and holy mosque visiting.[46] Perhaps expats involved in the study could explain the unawareness in part, but this has not been explored by the researchers.

Most EMS systems in the AGS are operated in the field by male staff with some minor exceptions such as the regional federal Dubai Ambulance Authority, which recently started with all-female first responder teams. Despite the female role in Dubai EMS, the rate of female to male is still low. In addition, most of the female providers are not citizens. A recent study in Dubai revealed that only one quarter of the participants are female and all of them were not Emirates. [47] Naturally, even when there are no female paramedics, women have full rights to use EMS. One included study showed that few participants still refuse all-male crews responding to female clients in the absence of her relatives.[28] This may be in part the cause that, on average, female clients with STEMI are late in attendance to emergency department compared to males in SA.[48] Therefore, women-centered research might give further insight into factors that prevent these women from accessing EMS. Furthermore, we found that male clients are the most prevalent consumers of EMS [12,15,16,18,20,29,30,32,34] To illustrate that, 48% of the AGS population are expats, and the majority of the expats are male.[46] As a consequence, the gender ratio in the AGS is considered to be 1 female to 2 males. Hence, the results related to the equity in this review are close to ratio of the real population in AGS.

Regarding ethnicity, the EMS in AGS provides services to all people with no discrimination. The studies included in this review showed that both citizens and non-citizens used EMS, but did not show sufficient data to explore differences in willingness to use EMS. Hence, further research is still needed to affirm the equity and accessibility to EMS.

Interestingly, one study indicated that low to moderate socio-economic status was a predictor of high EMS consumption.[16] It might be important for the high classes to be surveyed in future researches to identify factors affecting their willingness to use EMS in AGS.

### Limitations

The average quality of the included studies was only moderate. The apparent lack of good quality EMS research may be explained by the many difficulties faced worldwide.[49] For example, Delbridge, et al. demonstrated in their study the challenges and reasons for poor performance in conducting a high quality research in EMS in the US.[50] The last chapter of the IOM report described the challenges of optimizing the research that would help the American EMS. This chapter proposed an agenda to improve research, particularly interventional methods to improve the effectiveness.[51] Furthermore, none of the studies included in this review tested any predefined model to detect system improvement. An example of such a model is the one used in Singapore. They assessed their EMS system after they had applied a Discrete Event Simulation Model to detect the reduction of response time without any passive impact on the EMS utilization rate.[52]

## Conclusion

This review highlights the need for more and better studies of sufficient quality about all domains of quality in EMS in all the AGS. Future studies in urban and in rural areas should prioritize which cases are more crucial and therefore need to be handled more efficiently. EMS research in Kuwait and Bahrain is warranted as currently studies of EMS quality are unavailable for these States. Oman need further research to identify the possible causes for the low utilization rate for ambulance services for highly critical cases. Moreover, research into EMS efficiency should be conducted as not a single study was found on this domain.

## Supporting information

S1 Table Structure characteristic of EMS in the Arabian Gulf States. (DOCX)

S2 Table PRISMA 2009 checklist. (DOC)

S3 Table Search strategy for PubMed, web of science, CINAHL, and EMBASE up to June 1^st^, 2019. (DOCX)
