# Supplementary material for: What is known about the quality of out-of-hospital emergency medical services in the Arabian Gulf States? A systematic review
Source: PLoS One. 2019 Dec 19;14(12):e0226230. doi: 10.1371/journal.pone.0226230 (PMC6922377; doi:10.1371/journal.pone.0226230)
Supplement: S1 Table — (DOCX) [file pone.0226230.s001.docx]

**S1 Table 1. Structure Characteristic of EMS in the Arabian Gulf States.**

| **Country** | **Total Population**  **54.9**  **Million (M)** | **EMS Provider Organization:**   1. **Independent** 2. **Dependent** | **EMS:**   1. **1. Public** 2. **2. Private** 3. **3. Volunteer** | **1-EMS**  **Call Number**  **2-Free**  **of**  **Charge** | **EMS have**  **Independent**  **Call center** | **EMS Call**  **Center (CC)**  **operated**  **by** | **Process; From: Activation Emergency Call Number in CC; Through: Triaging Process in EMS Dispatch Room; To: Deploying Appropriate Ambulances to the Scene** | **Composition of EMS Dispatch**  **Center’s staff and Qualification who operate the Computer Aided Dispatch System** | **Type of**  **Ambulance**  **Crews and**  **Qualifications** | **TATV**   1. **AB** 2. **MICU** 3. **ALS** 4. **BLS**   **ANTRV: Y/N** |
| --- | --- | --- | --- | --- | --- | --- | --- | --- | --- | --- |
| **Saudi Arabia** | 32,6 M | **SRCA**   1. Independent | 1. Yes 2. No 3. No | 1. 997 2. Yes | Yes | **SRCA** | Calls are received, triaged, and dispatched in **CC** of **SRCA** by **SRCA**’s **EMT**s, and Paramedics and then they dispatch an appropriate ambulance to the scene | 1-Paramedic Call  Taker (Bachelor in **EMS**)  2-**EMT** Call Takers (Diploma in **EMS**)  3- Paramedic Dispatcher (Bachelor in **EMS**)  4- **EMT** Dispatchers (Diploma in **EMS**)  5- Physician **MD** as a Supervisor | 1-Physician **MD**  2-Paramedic  (Bachelor in **EMS**)  3-**EMT*** (Diploma in **EMS**)  4-Driver (license in **BLS** as first responder)) | 1. X 2. Yes; 3. Yes 4. No   **ANTRV**; Yes |
| **Oman** | 4,6 M | **PACDA**  2- Dependent on  **ROP** | 1. Yes 2. No 3. No | 1. 9999 2. Yes | No | **ROP** | Calls are received and screened by Policemen in **CC** of **ROP**, and then they pass it to **EMS** Dispatch Center in **PACDA** for triaging and **AS** dispatching to the scene | 1-**EMT** call Takers (Diploma in **EMS**)  2-**EMT** Dispatchers (Diploma in **EMS**) | 1-**EMT** (Diploma in **EMS**)  2-Driver (license in **BLS** as first responder) | 1. Yes 2. Yes; 3. Yes 4. No   **ANTRV**; No |
| **United Arab of Emirates**  **( UAE )** | 9,3 M | - **NAC** in north **UAE**   1- Independent   - **DCAS** in Dubai   1- Independent   - **AP** in Abu Dhabi   2- Dependent on police | 1. Yes, with **NAC** and **AP** 2. Yes, with **DCAS** 3. No | 1. 998 2. Yes | Yes | **DCAS**  **NAC**  **AP** | Based on client location in each region, calls are received, triaged and dispatched in **CC**s of **DCAS**, **NAC**, and **AP** by **EMT**s and Paramedics. They then dispatch an appropriate ambulance to the scene. | 1-Paramedic Call  Taker (Bachelor in **EMS**)  2-**EMT** Call Takers (Diploma in **EMS**)  3- Paramedic Dispatcher (Bachelor in **EMS**)  4- **EMT** Dispatchers (Diploma in **EMS**)  5- Physician **MD** as Supervisor | 1- Paramedic (Bachelor in **EMS/** Nursing)  2-**EMT** (Diploma in **EMS**)  3- Driver (license in **BLS** as first responder) | 1. Yes; 2. Yes; 3. Yes; 4. Yes;   **ANTRV**; Yes |
| **Qatar** | 2,7 M | **HMCAS**  1- Independent | 1. Yes 2. No 3. No | 1. 999 2. Yes | No | **MOI** | Calls are received and screened by Policemen in **CC** of **MOI,** and then they pass it to **EMS** Dispatch Center in **HMCAS** for triaging and **AS** dispatching to the scene | 1-Paramedic Call  Taker (Bachelor in **EMS**/ Nursing)  2-**EMT** Call Takers (Diploma in **EMS**)  3- Paramedic Dispatcher (Bachelor in **EMS**)  4- **EMT** Dispatchers (Diploma in **EMS**) | 1- Paramedic (Bachelor in **EMS** / Nursing)  2-**EMT** (Diploma in **EMS**) | 1. Yes 2. Yes 3. Yes 4. No   **ANTRV**; Yes |
| **Kuwait** | 4,2 M | **MOH**  1- Independent | 1. Yes 2. No 3. No | 1. 112 2. Yes | No | **MOI** | Calls are received and screened by Policemen in **CC** of **MOI** and then they pass it to **EMS** Dispatch Center in **MOH** for triaging and **AS** dispatching to the scene | 1-Paramedic call  Taker (Bachelor in **EM**S)  2-**EMT** call takers (Diploma in **EMS**)  3- Paramedic dispatcher (Bachelor in **EMS**)  4- **EMT** Dispatchers (Diploma in **EMS**) | 1- Paramedic (Bachelor in **EMS**)  2-EMT (Diploma in **EMS**)  3-Driver (license in **BLS** as first responder) | 1. X 2. Yes 3. Yes 4. No   **ANTRV**; **X** |
| **Bahrain** | 1,5 M | **MOH/**  **National**  **Ambulance**  **Project***  2- Dependent  on 3 Hospitals | 1. Yes 2. No 3. No | 1- 999  2- Yes | No | **MOI** | Calls are received and screened  by Policemen in **CC** of **MOI** and then they pass it to one of three Dispatch Centers in 3 different hospitals-based ambulances for triage and **AS** dispatching to scene | 1-Paramedic Call  Taker (Bachelor in **EMS**)  2-**EMT** Call Takers (Diploma in **EMS**)  3- Paramedic Dispatcher (Bachelor in **EMS**)  4- **EMT** Dispatchers (Diploma in **EMS**)  5- Physician **MD** as a Supervisor | 1-Paramedic  (Bachelor in **EMS**)  2- Staff Nurse (Bachelor in Nursing )  3- **EMT** (Diploma in **EMS**/ Nursing)  4- Driver (license in **BLS** as first responder)) | 1. **X** 2. Yes 3. Yes 4. Yes   **ANTRV**; No |

**AB;** Ambulance Buses, **ALS**; Advanced Life Support, **ANTRV**; Available non-transportation Response Vehicle, **AP**; Abu Dhabi Police, **AS**; Ambulance Services **BLS**; Basic Life Support, **CC**; Call Center, **DCAS**; Dubai Corporation for Ambulance Service **EMS**; Emergency Medical Services ***EMT**; Emergency Medical Technician: (i.e. a non-medical healthcare provider trained for at least two years to be competent on performing specific tasks in the out-of-hospital field for emergency condition of clients), **HMCAS**; Hamad Medical Cooperation Ambulance Services, **MD**; Medical Doctor **MICU**; Mobile Intensive Care Unit, **MOH**; Ministry of Health, **MOI**; Ministry of Interior **NAC**; National Ambulance Company, **PACDA**; Public Authority of Civil Defense and Ambulance, **ROP**; Royal Oman Police, **SRCA**; Saudi Red Crescent Authority, **TATV**; Type of Ambulance Transportation Vehicle, **X**; Unknown

* **National Ambulance Project:** This project has been recently launched in June 2019 and not yet completed.
