# Supplementary material for: What is known about the quality of out-of-hospital emergency medical services in the Arabian Gulf States? A systematic review
Source: PLoS One. 2019 Dec 19;14(12):e0226230. doi: 10.1371/journal.pone.0226230 (PMC6922377; doi:10.1371/journal.pone.0226230)
Supplement: S3 Table — (DOCX) [file pone.0226230.s003.docx]

**S3 Table 3. Search strategy for PubMed, web of Science, CINAHL, and EMBASE**

**up to June 1^st^, 2019**

| **Search strategy in PubMed: = 3032 hits)** | | |
| --- | --- | --- |
| Search #1 | "Ambulances"[MeSH] OR "Emergencies"[MeSH] OR "Emergency Medical Service Communication Systems"[MeSH] OR "Emergency Medical Services"[MeSH] OR "Emergency Medical Technicians"[MeSH] OR "Emergency Responders"[MeSH] OR "Emergency Treatment"[MeSH] OR "First aid"[MeSH] OR "Transportation of Patients"[MeSH] OR Accident and emergency[tiab] OR Ambulance provider*[tiab] OR Ambulance service*[tiab] OR Ambulance transport*[tiab] OR Ambulance transportation[tiab] OR Ambulance*[tiab] OR Emergencies[tiab] OR Emergency Care[tiab] OR Emergency department[tiab] OR Emergency dispatch[tiab] OR Emergency Health Service*[tiab] OR Emergency Medical Service*[tiab] OR Emergency Medical System[tiab] OR Emergency Medical Technician*[tiab] OR Emergency Mobile Unit*[tiab] OR Emergency Paramedic*[tiab] OR Emergency Personnel[tiab] OR Emergency Staff[tiab] OR Emergency treatment[tiab] OR Emergency Vehicle*[tiab] OR Emergicenter*[tiab] OR EMS Communication System*[tiab] OR First Aid[tiab] OR First responder*[tiab] OR Mobile Emergency Unit*[tiab] OR Non conveyance[tiab] OR Non-conveyance[tiab] OR "Out of hospital"[tiab] OR Paramedic*[tiab] OR Patient Conveyance[tiab] OR Patient transport*[tiab] OR Patients transport*[tiab] OR Pre Hospital[tiab] OR Prehospital[tiab] OR Pre-hospital[tiab] OR Prehospital care[tiab] OR Prehospital Emergency Care[tiab] OR Rescue worker*[tiab] OR Rescue[tiab] OR Transport Wounded and Sick[tiab] | Result #  380960 |
| Search #2 | "Accreditation"[MeSH] OR "Benchmarking"[MeSH] OR "Clinical Audit"[MeSH] OR "Clinical Competence"[MeSH] OR "Clinical Governance"[MeSH] OR "Efficiency"[MeSH] OR "Guideline Adherence"[MeSH] OR "Health Care Quality, Access, and Evaluation"[MeSH] OR "Health Equity"[MeSH] OR "Medical Errors"[MeSH] OR "Outcome and Process Assessment (Health Care)"[MeSH] OR "Outcome Assessment (Health Care)"[MeSH] OR "Patient Safety"[MeSH] OR "Patient Satisfaction"[MeSH] OR "Patient-Centered Care"[MeSH] OR "Process Assessment (Health Care)"[MeSH] OR "Quality Assurance, Health Care"[MeSH] OR "Risk Management"[MeSH] OR "Standard of Care"[MeSH] OR "Time Management"[MeSH] OR "Time Out, Healthcare"[MeSH] OR "Total Quality Management"[MeSH] OR "Work Performance"[MeSH] OR Accreditation*[tiab] OR Adverse Event*[tiab] OR Benchmark*[tiab] OR Best Practice*[tiab] OR Care standard*[tiab] OR Clinical audit*[tiab] OR Clinical Competenc*[tiab] OR Clinical Governance[tiab] OR Clinical Skill*[tiab] OR Donabedian model[tiab] OR Efficacy[tiab] OR Efficiency[tiab] OR Evaluation[tiab] OR Guideline adherence[tiab] OR Health equity[tiab] OR Incident Reporting[tiab] OR Medical Error*[tiab] OR Medical Mistake*[tiab] OR Outcome Assessment*[tiab] OR Patient centered[tiab] OR Patient safety[tiab] OR Patient Satisfaction[tiab] OR Performance indicator*[tiab] OR Performance measure*[tiab] OR Process Assessment*[tiab] OR Process Measure*[tiab] OR Productivity[tiab] OR Protocol Compliance[tiab] OR Quality[tiab] OR Risk management[tiab] OR Risks Management[tiab] OR Task performance[tiab] OR Time management[tiab] OR Time out[tiab] OR Time plan[tiab] OR Timeline[tiab] OR Utilisation[tiab] OR Utilization[tiab] OR Work performance[tiab] | Result #  8960296 |
| Search #3 | "Bahrain"[MeSH] OR "Kuwait"[MeSH] OR "Middle east"[MeSH] OR "Oman"[MeSH] OR "Qatar"[MeSH] OR "Saudi Arabia"[MeSH] OR "United Arab Emirates"[MeSH] OR Abha[tiab] OR Abu Dhabi[tiab] OR Ahmadi[tiab] OR Ajman[tiab] OR Al Ain[tiab] OR Al Baha[tiab] OR Al Batinah[tiab] OR Al Buraimi[tiab] OR Al Jawf[tiab] OR Al Khor[tiab] OR Al Madinah[tiab] OR Al Wakrah[tiab] OR Al Wusta[tiab] OR Al-Ahsa[tiab] OR Al-Qassim[tiab] OR Arabian Gulf Countries[tiab] OR Bahrain[tiab] OR Bahraini[tiab] OR Budaiya[tiab] OR Buraydah[tiab] OR Dammam[tiab] OR Dhofar[tiab] OR Doha[tiab] OR Dubai[tiab] OR Emirati[tiab] OR Fujairah[tiab] OR Gizan[tiab] OR Gulf Cooperation Council Countries[tiab] OR Hafar Al-Batin[tiab] OR Ha'il[tiab] OR Hamad[tiab] OR Hawalli[tiab] OR Isa Town[tiab] OR Jazan[tiab] OR Jeddah[tiab] OR Jizan[tiab] OR Jubail[tiab] OR Khamis Mushait[tiab] OR Khobar[tiab] OR Kuwait[tiab] OR Kuwaiti[tiab] OR Makkah[tiab] OR Manama[tiab] OR Mareb[tiab] OR Mecca[tiab] OR Medina[tiab] OR Middle east[tiab] OR Mubarak Al Kabeer[tiab] OR Muharraq[tiab] OR Musandam[tiab] OR Muscat[tiab] OR Najran[tiab] OR Oman[tiab] OR Omani[tiab] OR Qasim[tiab] OR Qatar[tiab] OR Qatari[tiab] OR Qatif[tiab] OR Ras Al-Khaimah[tiab] OR Riffa[tiab] OR Riyadh[tiab] OR Saudi Arabia[tiab] OR Saudi[tiab] OR Shabwah[tiab] OR Sharjah[tiab] OR Sitra[tiab] OR Tabuk[tiab] OR Ta'if[tiab] OR UAE[tiab] OR Umm Al Quwain[tiab] OR United Arab Emirates[tiab] OR Yanbu[tiab] | Result #  145484 |
| Search #4  article | **Search #1 AND Search #2 AND Search #3**  "Ambulances"[MeSH] OR "Emergencies"[MeSH] OR "Emergency Medical Service Communication Systems"[MeSH] OR "Emergency Medical Services"[MeSH] OR "Emergency Medical Technicians"[MeSH] OR "Emergency Responders"[MeSH] OR "Emergency Treatment"[MeSH] OR "First aid"[MeSH] OR "Transportation of Patients"[MeSH] OR Accident and emergency[tiab] OR Ambulance provider*[tiab] OR Ambulance service*[tiab] OR Ambulance transport*[tiab] OR Ambulance transportation[tiab] OR Ambulance*[tiab] OR Emergencies[tiab] OR Emergency Care[tiab] OR Emergency department[tiab] OR Emergency dispatch[tiab] OR Emergency Health Service*[tiab] OR Emergency Medical Service*[tiab] OR Emergency Medical System[tiab] OR Emergency Medical Technician*[tiab] OR Emergency Mobile Unit*[tiab] OR Emergency Paramedic*[tiab] OR Emergency Personnel[tiab] OR Emergency Staff[tiab] OR Emergency treatment[tiab] OR Emergency Vehicle*[tiab] OR Emergicenter*[tiab] OR EMS Communication System*[tiab] OR First Aid[tiab] OR First responder*[tiab] OR Mobile Emergency Unit*[tiab] OR Non conveyance[tiab] OR Non-conveyance[tiab] OR "Out of hospital"[tiab] OR Paramedic*[tiab] OR Patient Conveyance[tiab] OR Patient transport*[tiab] OR Patients transport*[tiab] OR Pre Hospital[tiab] OR Prehospital[tiab] OR Pre-hospital[tiab] OR Prehospital care[tiab] OR Prehospital Emergency Care[tiab] OR Rescue worker*[tiab] OR Rescue[tiab] OR Transport Wounded and Sick[tiab] AND "Accreditation"[MeSH] OR "Benchmarking"[MeSH] OR "Clinical Audit"[MeSH] OR "Clinical Competence"[MeSH] OR "Clinical Governance"[MeSH] OR "Efficiency"[MeSH] OR "Guideline Adherence"[MeSH] OR "Health Care Quality, Access, and Evaluation"[MeSH] OR "Health Equity"[MeSH] OR "Medical Errors"[MeSH] OR "Outcome and Process Assessment (Health Care)"[MeSH] OR "Outcome Assessment (Health Care)"[MeSH] OR "Patient Safety"[MeSH] OR "Patient Satisfaction"[MeSH] OR "Patient-Centered Care"[MeSH] OR "Process Assessment (Health Care)"[MeSH] OR "Quality Assurance, Health Care"[MeSH] OR "Risk Management"[MeSH] OR "Standard of Care"[MeSH] OR "Time Management"[MeSH] OR "Time Out, Healthcare"[MeSH] OR "Total Quality Management"[MeSH] OR "Work Performance"[MeSH] OR Accreditation*[tiab] OR Adverse Event*[tiab] OR Benchmark*[tiab] OR Best Practice*[tiab] OR Care standard*[tiab] OR Clinical audit*[tiab] OR Clinical Competenc*[tiab] OR Clinical Governance[tiab] OR Clinical Skill*[tiab] OR Donabedian model[tiab] OR Efficacy[tiab] OR Efficiency[tiab] OR Evaluation[tiab] OR Guideline adherence[tiab] OR Health equity[tiab] OR Incident Reporting[tiab] OR Medical Error*[tiab] OR Medical Mistake*[tiab] OR Outcome Assessment*[tiab] OR Patient centered[tiab] OR Patient safety[tiab] OR Patient Satisfaction[tiab] OR Performance indicator*[tiab] OR Performance measure*[tiab] OR Process Assessment*[tiab] OR Process Measure*[tiab] OR Productivity[tiab] OR Protocol Compliance[tiab] OR Quality[tiab] OR Risk management[tiab] OR Risks Management[tiab] OR Task performance[tiab] OR Time management[tiab] OR Time out[tiab] OR Time plan[tiab] OR Timeline[tiab] OR Utilisation[tiab] OR Utilization[tiab] OR Work performance[tiab] AND "Bahrain"[MeSH] OR "Kuwait"[MeSH] OR "Middle east"[MeSH] OR "Oman"[MeSH] OR "Qatar"[MeSH] OR "Saudi Arabia"[MeSH] OR "United Arab Emirates"[MeSH] OR Abha[tiab] OR Abu Dhabi[tiab] OR Ahmadi[tiab] OR Ajman[tiab] OR Al Ain[tiab] OR Al Baha[tiab] OR Al Batinah[tiab] OR Al Buraimi[tiab] OR Al Jawf[tiab] OR Al Khor[tiab] OR Al Madinah[tiab] OR Al Wakrah[tiab] OR Al Wusta[tiab] OR Al-Ahsa[tiab] OR Al-Qassim[tiab] OR Arabian Gulf Countries[tiab] OR Bahrain[tiab] OR Bahraini[tiab] OR Budaiya[tiab] OR Buraydah[tiab] OR Dammam[tiab] OR Dhofar[tiab] OR Doha[tiab] OR Dubai[tiab] OR Emirati[tiab] OR Fujairah[tiab] OR Gizan[tiab] OR Gulf Cooperation Council Countries[tiab] OR Hafar Al-Batin[tiab] OR Ha'il[tiab] OR Hamad[tiab] OR Hawalli[tiab] OR Isa Town[tiab] OR Jazan[tiab] OR Jeddah[tiab] OR Jizan[tiab] OR Jubail[tiab] OR Khamis Mushait[tiab] OR Khobar[tiab] OR Kuwait[tiab] OR Kuwaiti[tiab] OR Makkah[tiab] OR Manama[tiab] OR Mareb[tiab] OR Mecca[tiab] OR Medina[tiab] OR Middle east[tiab] OR Mubarak Al Kabeer[tiab] OR Muharraq[tiab] OR Musandam[tiab] OR Muscat[tiab] OR Najran[tiab] OR Oman[tiab] OR Omani[tiab] OR Qasim[tiab] OR Qatar[tiab] OR Qatari[tiab] OR Qatif[tiab] OR Ras Al-Khaimah[tiab] OR Riffa[tiab] OR Riyadh[tiab] OR Saudi Arabia[tiab] OR Saudi[tiab] OR Shabwah[tiab] OR Sharjah[tiab] OR Sitra[tiab] OR Tabuk[tiab] OR Ta'if[tiab] OR UAE[tiab] OR Umm Al Quwain[tiab] OR United Arab Emirates[tiab] OR Yanbu[tiab] | Result #  3032 |

| **Search strategy in Web of Science: up to 01 June (n= 333 hits)** | | |
| --- | --- | --- |
| Search #1 | TS= "Accident and emergency" OR TS= "Ambulance provider*" OR TS= "Ambulance service*" OR TS= "Ambulance transport*" OR TS= "Ambulance transportation" OR TS= "Emergency Care" OR TS= "Emergency department" OR TS= "Emergency dispatch" OR TS= "Emergency Health Service*" OR TS= "Emergency Medical Service Communication Systems" OR TS= "Emergency Medical Service*" OR TS= "Emergency Medical System" OR TS= "Emergency Medical Technician*" OR TS= "Emergency Mobile Unit*" OR TS= "Emergency Paramedic*" OR TS= "Emergency Personnel" OR TS= "Emergency Responders" OR TS= "Emergency Staff" OR TS= "Emergency Treatment" OR TS= "Emergency Vehicle*" OR TS= "EMS Communication System*" OR TS= "First aid" OR TS= "First responder*" OR TS= "Mobile Emergency Unit*" OR TS= "Non conveyance" OR TS= "Out of hospital" OR TS= "Patient Conveyance" OR TS= "Patient transport*" OR TS= "Patients transport*" OR TS= "Pre Hospital" OR TS= "Prehospital care" OR TS= "Prehospital Emergency Care" OR TS= "Rescue worker*" OR TS= "Transport Wounded and Sick" OR TS= "Transportation of Patients" OR TS= Ambulance* OR TS= Emergencies OR TS= Emergicenter* OR TS= Non-conveyance OR TS= Paramedic* OR TS= Pre-hospital OR TS= Prehospital OR TS= Rescue | Result #  353,058 |
| Search #2 | TS= "Adverse Event*" OR TS= "Best Practice*" OR TS= "Care standard*" OR TS= "Clinical Audit*" OR TS= "Clinical Competenc*" OR TS= "Clinical Governance" OR TS= "Clinical Skill*" OR TS= "Donabedian model" OR TS= "Guideline Adherence" OR TS= "Health Care Quality, Access, and Evaluation" OR TS= "Health Equity" OR TS= "Incident Reporting" OR TS= "Medical Error*" OR TS= "Outcome and Process Assessment (Health Care)" OR TS= "Outcome Assessment (Health Care)" OR TS= "Outcome Assessment*" OR TS= "Patient centered" OR TS= "Patient Safety" OR TS= "Patient Satisfaction" OR TS= "Patient-Centered Care" OR TS= "Performance indicator*" OR TS= "Performance measure*" OR TS= "Process Assessment (Health Care)" OR TS= "Process Assessment*" OR TS= "Process Measure*" OR TS= "Protocol Compliance" OR TS= "Quality Assurance, Health Care" OR TS= "Risk Management" OR TS= "Risk management" OR TS= "Standard of Care" OR TS= "Task performance" OR TS= "Time Management" OR TS= "Time out" OR TS= "Time Out, Healthcare" OR TS= "Time plan" OR TS= "Total Quality Management" OR TS= "Work Performance" OR TS= Accreditation* OR TS= Benchmark* OR TS= Benchmarkin OR TS= Efficacy OR TS= Efficiency OR TS= Evaluation OR TS= Productivity OR TS= Quality OR TS= Timeline OR TS= Utilisation OR TS= Utilization | Result #  5,673,871 |
| Search #3 | TS= Abha OR TS= Abu Dhabi OR TS= Ahmadi OR TS= Ajman OR TS= Al Ain OR TS= Al Baha OR TS= Al Batinah OR TS= Al Buraimi OR TS= Al Jawf OR TS= Al Kabeer OR TS= Al Khor OR TS= Al Madinah OR TS= Al Wakrah OR TS= Al Wusta OR TS= Al-Ahsa OR TS= Al-Qassim OR TS= Arabian Gulf Countries OR TS= Asir OR TS= Bahrain OR TS= Bahraini OR TS= Budaiya OR TS= Buraydah OR TS= Dammam OR TS= Dhofar OR TS= Doha OR TS= Dubai OR TS= Emirati OR TS= Fujairah OR TS= Gizan OR TS= Gulf Cooperation Council Countries OR TS= Hafar Al-Batin OR TS= Ha'il OR TS= Hamad OR TS= Hawalli OR TS= Isa Town OR TS= Jazan OR TS= Jeddah OR TS= Jizan OR TS= Jubail OR TS= Khamis Mushait OR TS= Khobar OR TS= Kuwait OR TS= Kuwaiti OR TS= Makkah OR TS= Manama OR TS= Mareb OR TS= Mecca OR TS= Medina OR TS= Middle east OR TS= Mubarak OR TS= Muharraq OR TS= Musandam OR TS= Muscat OR TS= Najran OR TS= Oman OR TS= Omani OR TS= Qasim OR TS= Qatar OR TS= Qatari OR TS= Qatif OR TS= Ras Al-Khaimah OR TS= Riffa OR TS= Riyadh OR TS= Saudi OR TS= Saudi Arabia OR TS= Shabwah OR TS= Sharjah OR TS= Sitra OR TS= Tabuk OR TS= Ta'if OR TS= UAE OR TS= Umm Al Quwain OR TS= United Arab Emirates OR TS= Yanbu | Result #  101,949 |
| Search #4 | **Search #1 AND Search #2 AND Search #3**  (TS= "Accident and emergency" OR TS= "Ambulance provider*" OR TS= "Ambulance service*" OR TS= "Ambulance transport*" OR TS= "Ambulance transportation" OR TS= "Emergency Care" OR TS= "Emergency department" OR TS= "Emergency dispatch" OR TS= "Emergency Health Service*" OR TS= "Emergency Medical Service Communication Systems" OR TS= "Emergency Medical Service*" OR TS= "Emergency Medical System" OR TS= "Emergency Medical Technician*" OR TS= "Emergency Mobile Unit*" OR TS= "Emergency Paramedic*" OR TS= "Emergency Personnel" OR TS= "Emergency Responders" OR TS= "Emergency Staff" OR TS= "Emergency Treatment" OR TS= "Emergency Vehicle*" OR TS= "EMS Communication System*" OR TS= "First aid" OR TS= "First responder*" OR TS= "Mobile Emergency Unit*" OR TS= "Non conveyance" OR TS= "Out of hospital" OR TS= "Patient Conveyance" OR TS= "Patient transport*" OR TS= "Patients transport*" OR TS= "Pre Hospital" OR TS= "Prehospital care" OR TS= "Prehospital Emergency Care" OR TS= "Rescue worker*" OR TS= "Transport Wounded and Sick" OR TS= "Transportation of Patients" OR TS= Ambulance* OR TS= Emergencies OR TS= Emergicenter* OR TS= Non-conveyance OR TS= Paramedic* OR TS= Pre-hospital OR TS= Prehospital OR TS= Rescue) AND (TS= "Adverse Event*" OR TS= "Best Practice*" OR TS= "Care standard*" OR TS= "Clinical Audit*" OR TS= "Clinical Competenc*" OR TS= "Clinical Governance" OR TS= "Clinical Skill*" OR TS= "Donabedian model" OR TS= "Guideline Adherence" OR TS= "Health Care Quality, Access, and Evaluation" OR TS= "Health Equity" OR TS= "Incident Reporting" OR TS= "Medical Error*" OR TS= "Outcome and Process Assessment (Health Care)" OR TS= "Outcome Assessment (Health Care)" OR TS= "Outcome Assessment*" OR TS= "Patient centered" OR TS= "Patient Safety" OR TS= "Patient Satisfaction" OR TS= "Patient-Centered Care" OR TS= "Performance indicator*" OR TS= "Performance measure*" OR TS= "Process Assessment (Health Care)" OR TS= "Process Assessment*" OR TS= "Process Measure*" OR TS= "Protocol Compliance" OR TS= "Quality Assurance, Health Care" OR TS= "Risk Management" OR TS= "Risk management" OR TS= "Standard of Care" OR TS= "Task performance" OR TS= "Time Management" OR TS= "Time out" OR TS= "Time Out, Healthcare" OR TS= "Time plan" OR TS= "Total Quality Management" OR TS= "Work Performance" OR TS= Accreditation* OR TS= Benchmark* OR TS= Benchmarkin OR TS= Efficacy OR TS= Efficiency OR TS= Evaluation OR TS= Productivity OR TS= Quality OR TS= Timeline OR TS= Utilisation OR TS= Utilization) AND (TS= Abha OR TS= Abu Dhabi OR TS= Ahmadi OR TS= Ajman OR TS= Al Ain OR TS= Al Baha OR TS= Al Batinah OR TS= Al Buraimi OR TS= Al Jawf OR TS= Al Kabeer OR TS= Al Khor OR TS= Al Madinah OR TS= Al Wakrah OR TS= Al Wusta OR TS= Al-Ahsa OR TS= Al-Qassim OR TS= Arabian Gulf Countries OR TS= Asir OR TS= Bahrain OR TS= Bahraini OR TS= Budaiya OR TS= Buraydah OR TS= Dammam OR TS= Dhofar OR TS= Doha OR TS= Dubai OR TS= Emirati OR TS= Fujairah OR TS= Gizan OR TS= Gulf Cooperation Council Countries OR TS= Hafar Al-Batin OR TS= Ha'il OR TS= Hamad OR TS= Hawalli OR TS= Isa Town OR TS= Jazan OR TS= Jeddah OR TS= Jizan OR TS= Jubail OR TS= Khamis Mushait OR TS= Khobar OR TS= Kuwait OR TS= Kuwaiti OR TS= Makkah OR TS= Manama OR TS= Mareb OR TS= Mecca OR TS= Medina OR TS= Middle east OR TS= Mubarak OR TS= Muharraq OR TS= Musandam OR TS= Muscat OR TS= Najran OR TS= Oman OR TS= Omani OR TS= Qasim OR TS= Qatar OR TS= Qatari OR TS= Qatif OR TS= Ras Al-Khaimah OR TS= Riffa OR TS= Riyadh OR TS= Saudi OR TS= Saudi Arabia OR TS= Shabwah OR TS= Sharjah OR TS= Sitra OR TS= Tabuk OR TS= Ta'if OR TS= UAE OR TS= Umm Al Quwain OR TS= United Arab Emirates OR TS= Yanbu) | Result #  333 |

| **Search strategy in CINAHL: (n= 167 hits)** | | |
| --- | --- | --- |
| Search #1 | (MH "Emergency Medical Service Communication Systems") OR (MH "Emergency Service Information Systems") OR (MH "Emergency Medical Services") OR(MH "Emergency Medicine") OR (MH "Emergency Service") OR (MH "Emergency Medical Technician Attitudes") OR ""Emergency Medical Service Communication Systems"" OR (MH "Emergency Medical Technicians") OR ""Emergency Medical Services"" OR ""Emergency Medical Technicians"" OR (MH "Emergency Patients") OR ""Emergency Responders"" OR (MH "Transportation of Patients") OR ""Transportation of Patients"" OR ""Accident and emergency"" OR "Ambulance provider*" OR "Ambulance service*" OR "Ambulance transport*" OR "Ambulance transportation" OR "Ambulance*" OR (MH "Ambulation Aids") OR "Emergencies" OR (MH "Emergencies") OR (MH "Emergency Care") OR "Emergency Care" OR "Emergency department" OR "Emergency dispatch" OR "Emergency Health Service*" OR "Emergency Medical System" OR "Emergency Medical Technician*" OR "Emergency Mobile Unit*" OR "Emergency Paramedic*" OR "Emergency Personnel" OR "Emergency Staff" OR (MH "Emergency Treatment") OR "Emergency treatment" OR "Emergency Vehicle*" OR "Emergicenter*" OR "EMS Communication System*" OR (MH "First Aid") OR "First Aid" OR "First responder*" OR "Mobile Emergency Unit*" OR "Non conveyance" OR "Non-conveyance" OR (MH "Bystander CPR") OR ""Out of hospital"" OR "Paramedic*" OR "Patient Conveyance" OR (MH "Transportation of Patients+/UT") OR "Patient transport*" OR "Patients transport*" OR "Pre Hospital" OR (MH "Prehospital Care") OR "Prehospital" OR (MH "Rapid Response (Emergency Care)/UT/TD/ST/OG/EV/ES/CT/CL/AE") OR "Pre-hospital" OR "Prehospital care" OR "Prehospital Emergency Care" OR "Rescue worker*" OR (MH "Rescue Work/UT/TD/EV/AM/CL/MA/ST") OR "Rescue" OR ""Transport Wounded and Sick"" | Result #  166,394 |
| Search #2 | ""Health Care Quality, Access, and Evaluation"" OR ""Outcome and Process Assessment (Health Care)"" OR ""Patient-Centered Care"" OR ""Process Assessment (Health Care)"" OR ""Quality Assurance, Health Care"" OR ""Standard of Care"" OR ""Time Out, Healthcare"" OR ""Total Quality Management"" OR "Accreditation*" OR "Adverse Event*" OR "Benchmark*" OR "Best Practice*" OR "Care standard*" OR "Clinical audit*" OR "Clinical Competenc*" OR "Clinical Governance" OR "Clinical Skill*" OR "Donabedian model" OR "Efficacy" OR "Efficiency" OR "Guideline adherence" OR "Health equity" OR "Incident Reporting" OR "Medical Error*" OR "Outcome Assessment*" OR "Patient centered" OR "Patient safety" OR "Patient Satisfaction" OR "Patients Satisfaction" OR "Performance indicator*" OR "Performance measure*" OR "Productivity" OR "Protocol Compliance" OR "Quality" OR "Risk management" OR "Risks Management" OR "Task performance" OR "Time management" OR "Time out" OR "Time plan" OR "Timeline" OR "Utilisation" OR "Utilization" OR "Work Performance" OR (MH "Adverse Drug Event") OR (MH "Adverse Health Care Event")OR (MH "Attitude of Health Personnel") OR (MH "Attitude to Risk") OR (MH "Benchmarking") OR (MH "Clinical Assessment Tools") OR (MH "Clinical Competence") OR (MH "Clinical Governance") OR (MH "Clinical Indicators") OR (MH "Critical Incident Stress") OR (MH "Critical Incidents Method") OR (MH "Decision Support Systems, Clinical") OR (MH "Evaluation and Quality Improvement Program") OR (MH "Evaluation Research") OR (MH "Evaluation") OR (MH "Guideline Adherence") OR (MH "Health Care Delivery") OR (MH "Health Care Delivery, Integrated") OR (MH "Health Care Errors") OR (MH "Health Care Reform") OR (MH "Health Resource Utilization") OR (MH "Health Services Accessibility") OR (MH "Incident Reports") OR (MH "Job Performance") OR (MH "Mandatory Reporting") OR (MH "Mass Casualty Incidents") OR (MH "Outcome Assessment") OR (MH "Outcomes (Health Care)") OR (MH "Patient Centered Care") OR (MH "Patient Compliance") OR (MH "Patient Safety") OR (MH "Patient Satisfaction") OR (MH "Patient-Reported Outcomes") OR (MH "Performance Measurement Systems") OR (MH "Personnel Management") OR (MH "Physical Performance") OR (MH "Practice Guidelines") OR (MH "Practice Patterns") OR (MH "Process Assessment (Health Care)") OR (MH "Productivity") OR (MH "Protocols") OR (MH "Quality Circles") OR (MH "Quality Control (Technology)") OR (MH "Quality Improvement") OR (MH "Quality Management, Organizational") OR (MH "Quality of Health Care") OR (MH "Risk Management") OR (MH "Self-Efficacy") OR (MH "Task Performance and Analysis") OR (MH "Time Factors") OR (MH "Time Management") OR (MH "Treatment Errors") OR (MH "Turnaround Time") OR (MH "Utilization Review") OR (MH "Voluntary Reporting") OR(MH "Work Environment") OR (MH "Work Redesign") | Result #  1,546,939 |
| Search #3 | Abha OR "Abu Dhabi" OR Ahmadi OR Ajman OR "Al Ain" OR "Al baha" OR "Al Batinah" OR "Al Buraimi" OR "Al Jawf" OR "Al Kabeer" OR "Al Khor" OR "Al Madinah" OR "Al Wakrah" OR "Al Wusta" OR Al-Ahsa OR Al-Qassim OR "Arabian Gulf Countries" OR Asir OR Bahrain OR Bahraini OR Budaiya OR Buraydah OR Dammam OR Dhofar OR Doha OR Dubai OR Emirati OR Fujairah OR Gizan OR "Gulf Cooperation Council Countries" OR Hafar Al-Batin ORHa'il OR Hamad OR Hawalli OR Isa Town OR Jazan OR Jeddah OR Jizan OR Jubail OR "Khamis Mushait" OR Khobar OR Kuwait OR Kuwaiti OR Makkah OR Manama OR Mareb OR Mecca OR Medina OR (MH "Saudi Arabia") OR (MH "Qatar") OR (MH "Kuwait") OR (MH "United Arab Emirates") OR (MH "Bahrain") OR (MH "Oman") OR Mubarak OR Muharraq OR Musandam OR Muscat OR Najran OR Oman OR Omani OR Qasim OR Qatar OR Qatari OR Qatif OR "Ras Al-Khaimah" OR Riffa OR Riyadh OR Saudi OR "Saudi Arabia" OR Shabwah OR Sharjah OR Sitra OR Tabuk OR Ta'if OR UAE OR "Umm Al Quwain" OR "United Arab Emirates" OR Yanbu | Result #  10,463 |
|  | **Search #1 AND Search #2 AND Search #3**  ((MH "Emergency Medical Service Communication Systems") OR (MH "Emergency Service Information Systems") OR (MH "Emergency Medical Services") OR(MH "Emergency Medicine") OR (MH "Emergency Service") OR (MH "Emergency Medical Technician Attitudes") OR ""Emergency Medical Service Communication Systems"" OR (MH "Emergency Medical Technicians") OR ""Emergency Medical Services"" OR ""Emergency Medical Technicians"" OR (MH "Emergency Patients") OR ""Emergency Responders"" OR (MH "Transportation of Patients") OR ""Transportation of Patients"" OR ""Accident and emergency"" OR "Ambulance provider*" OR "Ambulance service*" OR "Ambulance transport*" OR "Ambulance transportation" OR "Ambulance*" OR (MH "Ambulation Aids") OR "Emergencies" OR (MH "Emergencies") OR (MH "Emergency Care") OR "Emergency Care" OR "Emergency department" OR "Emergency dispatch" OR "Emergency Health Service*" OR "Emergency Medical System" OR "Emergency Medical Technician*" OR "Emergency Mobile Unit*" OR "Emergency Paramedic*" OR "Emergency Personnel" OR "Emergency Staff" OR (MH "Emergency Treatment") OR "Emergency treatment" OR "Emergency Vehicle*" OR "Emergicenter*" OR "EMS Communication System*" OR (MH "First Aid") OR "First Aid" OR "First responder*" OR "Mobile Emergency Unit*" OR "Non conveyance" OR "Non-conveyance" OR (MH "Bystander CPR") OR ""Out of hospital"" OR "Paramedic*" OR "Patient Conveyance" OR (MH "Transportation of Patients+/UT") OR "Patient transport*" OR "Patients transport*" OR "Pre Hospital" OR (MH "Prehospital Care") OR "Prehospital" OR (MH "Rapid Response (Emergency Care)/UT/TD/ST/OG/EV/ES/CT/CL/AE") OR "Pre-hospital" OR "Prehospital care" OR "Prehospital Emergency Care" OR "Rescue worker*" OR (MH "Rescue Work/UT/TD/EV/AM/CL/MA/ST") OR "Rescue" OR ""Transport Wounded and Sick"") AND (""Health Care Quality, Access, and Evaluation"" OR ""Outcome and Process Assessment (Health Care)"" OR ""Patient-Centered Care"" OR ""Process Assessment (Health Care)"" OR ""Quality Assurance, Health Care"" OR ""Standard of Care"" OR ""Time Out, Healthcare"" OR ""Total Quality Management"" OR "Accreditation*" OR "Adverse Event*" OR "Benchmark*" OR "Best Practice*" OR "Care standard*" OR "Clinical audit*" OR "Clinical Competenc*" OR "Clinical Governance" OR "Clinical Skill*" OR "Donabedian model" OR "Efficacy" OR "Efficiency" OR "Guideline adherence" OR "Health equity" OR "Incident Reporting" OR "Medical Error*" OR "Outcome Assessment*" OR "Patient centered" OR "Patient safety" OR "Patient Satisfaction" OR "Patients Satisfaction" OR "Performance indicator*" OR "Performance measure*" OR "Productivity" OR "Protocol Compliance" OR "Quality" OR "Risk management" OR "Risks Management" OR "Task performance" OR "Time management" OR "Time out" OR "Time plan" OR "Timeline" OR "Utilisation" OR "Utilization" OR "Work Performance" OR (MH "Adverse Drug Event") OR (MH "Adverse Health Care Event")OR (MH "Attitude of Health Personnel") OR (MH "Attitude to Risk") OR (MH "Benchmarking") OR (MH "Clinical Assessment Tools") OR (MH "Clinical Competence") OR (MH "Clinical Governance") OR (MH "Clinical Indicators") OR (MH "Critical Incident Stress") OR (MH "Critical Incidents Method") OR (MH "Decision Support Systems, Clinical") OR (MH "Evaluation and Quality Improvement Program") OR (MH "Evaluation Research") OR (MH "Evaluation") OR (MH "Guideline Adherence") OR (MH "Health Care Delivery") OR (MH "Health Care Delivery, Integrated") OR (MH "Health Care Errors") OR (MH "Health Care Reform") OR (MH "Health Resource Utilization") OR (MH "Health Services Accessibility") OR (MH "Incident Reports") OR (MH "Job Performance") OR (MH "Mandatory Reporting") OR (MH "Mass Casualty Incidents") OR (MH "Outcome Assessment") OR (MH "Outcomes (Health Care)") OR (MH "Patient Centered Care") OR (MH "Patient Compliance") OR (MH "Patient Safety") OR (MH "Patient Satisfaction") OR (MH "Patient-Reported Outcomes") OR (MH "Performance Measurement Systems") OR (MH "Personnel Management") OR (MH "Physical Performance") OR (MH "Practice Guidelines") OR (MH "Practice Patterns") OR (MH "Process Assessment (Health Care)") OR (MH "Productivity") OR (MH "Protocols") OR (MH "Quality Circles") OR (MH "Quality Control (Technology)") OR (MH "Quality Improvement") OR (MH "Quality Management, Organizational") OR (MH "Quality of Health Care") OR (MH "Risk Management") OR (MH "Self-Efficacy") OR (MH "Task Performance and Analysis") OR (MH "Time Factors") OR (MH "Time Management") OR (MH "Treatment Errors") OR (MH "Turnaround Time") OR (MH "Utilization Review") OR (MH "Voluntary Reporting") OR(MH "Work Environment") OR (MH "Work Redesign")) AND (Abha OR "Abu Dhabi" OR AhmadiOR Ajman OR "Al Ain" OR "Al baha" OR "Al Batinah" OR "Al Buraimi" OR "Al Jawf" OR "Al Kabeer" OR "Al Khor" OR "Al Madinah" OR "Al Wakrah" OR "Al Wusta" OR Al-Ahsa OR Al-Qassim OR "Arabian Gulf Countries" OR Asir OR Bahrain OR Bahraini OR Budaiya OR Buraydah OR Dammam OR Dhofar OR Doha OR Dubai OR Emirati OR Fujairah OR Gizan OR "Gulf Cooperation Council Countries" OR Hafar Al-Batin ORHa'il OR Hamad OR Hawalli OR Isa Town OR Jazan OR Jeddah OR Jizan OR Jubail OR "Khamis Mushait" OR Khobar OR Kuwait OR Kuwaiti OR Makkah OR Manama OR Mareb OR Mecca OR Medina OR (MH "Saudi Arabia") OR (MH "Qatar") OR (MH "Kuwait") OR (MH "United Arab Emirates") OR (MH "Bahrain") OR (MH "Oman") OR Mubarak OR Muharraq OR Musandam OR Muscat OR Najran OR Oman OR Omani OR Qasim OR Qatar OR Qatari OR Qatif OR "Ras Al-Khaimah" OR Riffa OR Riyadh OR Saudi OR "Saudi Arabia" OR Shabwah OR Sharjah OR Sitra OR Tabuk OR Ta'if OR UAE OR "Umm Al Quwain" OR "United Arab Emirates" OR Yanbu OR "Kingdom of Bahrain") | Result #  167 |

| **Search strategy in Embase: (n= 1694 hits)** | | |
| --- | --- | --- |
| Search #1 | Accident and emergency.mp. OR Ambulance provider*.mp. OR Ambulance service*.mp. OR Ambulance transport*.mp. OR Ambulance*.mp. OR Emergencies.mp. OR Emergency Care.mp. OR Emergency department.mp. OR Emergency dispatch.mp. OR Emergency Medical Service Communication Systems.mp. OR Emergency Medical Service*.mp. OR Emergency Medical System.mp. OR Emergency Medical Technician*.mp. OR Emergency Medical Technicians.mp. OR Emergency Mobile Unit*.mp. OR Emergency Paramedic*.mp. OR Emergency Personnel.mp. OR Emergency Responders.mp. OR Emergency Staff.mp. OR Emergency Treatment.mp. OR Emergency Vehicle*.mp. OR Emergency.mp. OR Emergicenter*.mp. OR EMS Communication System*.mp. OR exp "out of hospital cardiac arrest"/ OR exp ambulance diversion/ OR exp ambulance response time/ OR exp ambulance transportation/ OR exp ambulance/ OR exp automated external defibrillator/ OR exp defibrillator/ OR exp emergency call system/ OR exp emergency care/ OR exp emergency health service/ OR exp emergency medical dispatch/ OR exp emergency medical dispatcher/ OR exp emergency medicine/ OR exp emergency patient/ OR exp emergency treatment/ OR exp emergency/ OR exp first aid/ OR exp intensive care/ OR exp paramedical personnel/ OR exp patient transport/ OR exp rescue personnel/ OR exp rescue work/ OR First aid.mp. OR First responder*.mp. OR Mobile Emergency Unit*.mp. OR Non conveyance.mp. OR Non-conveyance.mp. OR Out of hospital.mp. OR Paramedic*.mp. OR Patient Conveyance.mp. OR Patient transport*.mp. OR Patients transport*.mp. OR Pre Hospital.mp. OR Prehospital care.mp. OR Prehospital Emergency Care.mp. OR Prehospital.mp. OR Pre-hospital.mp. OR Rescue worker*.mp. OR Rescue.mp. OR Transportation of Patients.mp. | Result #  1688855 |
| Search #2 | Accreditation.mp. OR Adverse Event*.mp. OR Benchmark*.mp. OR Care standard*.mp. OR Clinical Audit.mp. OR Clinical Governance.mp. OR Donabedian model.mp. OR Efficacy.mp. OR Efficiency.mp. OR Evaluation.mp. OR exp accident prevention/ OR exp accreditation/ OR exp benchmarking/ OR exp clinical audit/ OR exp clinical competence/ OR exp clinical practice/ OR exp decision making/ OR exp health care quality/ OR exp health equity/ OR exp incident report/ OR exp job performance/ OR exp malpractice/ OR exp management/ OR exp medical audit/ OR exp medical error/ OR exp outcome assessment/ OR exp outcome assessment/ OR exp patient care/ OR exp patient safety/ OR exp patient satisfaction/ OR exp performance measurement system/ OR exp practice guideline/ OR exp productivity/ OR exp protocol compliance/ OR exp quality control procedures/ OR exp quality control/ OR exp risk management/ OR exp standard/ OR exp task performance/ OR exp time management/ OR exp time out/ OR exp time/ OR exp total quality management/ OR exp treatment outcome/ OR Guideline Adherence.mp. OR Health Care Quality, Access, and Evaluation.mp. OR Health Equity.mp. OR Incident Reporting.mp. OR Medical Error*.mp. OR Medical Error*.mp. OR Medical Mistake*.mp. OR "Outcome and Process Assessment (Health Care)".mp. OR "Outcome Assessment (Health Care)".mp. OR Outcome Assessment*.mp. OR Patient centered.mp. OR Patient Safety.mp. OR Patient Satisfaction.mp. OR Patient-Centered Care.mp. OR Performance indicator*.mp. OR Performance measure*.mp. OR "Process Assessment (Health Care)".mp. OR Process Assessment*.mp. OR Process Measure*.mp. OR Productivity.mp. OR Protocol Compliance.mp. OR Quality Assurance, Health Care.mp. OR Quality.mp. OR Risk Management.mp. OR Risks Management.mp. OR Standard of Care.mp. OR Task performance.mp. OR Time Management.mp. OR Time Out, Healthcare.mp. OR Time out.mp. OR Time plan.mp. OR Timeline.mp. OR Total Quality Management.mp. OR Utilisation.mp. OR Utilization.mp. OR Work Performance.mp. | Result #  4411464 |
| Search #3 | Abha.mp. OR Abu Dhabi.mp. OR Ahmadi.mp. OR Ajman.mp. OR Al Ain.mp. OR Al baha OR Al Batinah.mp. OR Al Buraimi.mp. OR Al Jawf.mp. OR Al Khor.mp. OR Al Madinah.mp. OR Al Wakrah.mp. OR Al Wusta.mp. OR Al-Ahsa.mp. OR Al-Qassim.mp. OR Arabian Gulf Countries.mp. OR Asir.mp. OR Bahrain.mp. OR Bahraini.mp. OR Budaiya.mp. OR Buraydah.mp. OR Dammam.mp. OR Dhofar.mp. OR Doha.mp. OR Dubai.mp. OR Emirati.mp. OR exp Bahrain/ OR Exp Kuwait/ OR Exp Oman/ OR Exp Qatar/ OR Exp Saudi Arabia/ OR Exp United Arab Emirates/ OR Fujairah.mp. OR Gizan.mp. OR Gulf Cooperation Council Countries.mp. OR Hafar Al-Batin.mp. OR Ha'il.mp. OR Hamad.mp. OR Hawalli.mp. OR Isa Town.mp. OR Jazan.mp. OR Jeddah.mp. OR Jizan.mp. OR Jubail.mp. OR Khamis Mushait.mp. OR Khobar.mp. OR Kuwait.mp. OR Kuwaiti.mp. OR Makkah.mp. OR Manama.mp. OR Mareb.mp. OR Mecca.mp. OR Medina.mp. OR Middle east/ OR Middle east.mp. OR Mubarak Al Kabeer.mp. OR Muharraq.mp. OR Musandam.mp. OR Muscat.mp. OR Najran.mp. OR Oman.mp. OR Omani.mp. OR Qasim.mp. OR Qatar.mp. OR Qatari.mp. OR Qatif.mp. OR Ras Al-Khaimah.mp. OR Riffa.mp. OR Riyadh.mp. OR Saudi Arabia.mp. OR Saudi.mp. OR Shabwah.mp. OR Sharjah.mp. OR Sitra.mp. OR Tabuk.mp. OR Ta'if.mp. OR UAE.mp. OR Umm Al Quwain.mp. OR United Arab Emirates.mp. OR Yanbu.mp. | Result #  65367 |
|  | **Search #1 AND Search #2 AND Search #3**  (Accident and emergency.mp. OR Ambulance provider*.mp. OR Ambulance service*.mp. OR Ambulance transport*.mp. OR Ambulance*.mp. OR Emergencies.mp. OR Emergency Care.mp. OR Emergency department.mp. OR Emergency dispatch.mp. OR Emergency Medical Service Communication Systems.mp. OR Emergency Medical Service*.mp. OR Emergency Medical System.mp. OR Emergency Medical Technician*.mp. OR Emergency Medical Technicians.mp. OR Emergency Mobile Unit*.mp. OR Emergency Paramedic*.mp. OR Emergency Personnel.mp. OR Emergency Responders.mp. OR Emergency Staff.mp. OR Emergency Treatment.mp. OR Emergency Vehicle*.mp. OR Emergency.mp. OR Emergicenter*.mp. OR EMS Communication System*.mp. OR exp "out of hospital cardiac arrest"/ OR exp ambulance diversion/ OR exp ambulance response time/ OR exp ambulance transportation/ OR exp ambulance/ OR exp automated external defibrillator/ OR exp defibrillator/ OR exp emergency call system/ OR exp emergency care/ OR exp emergency health service/ OR exp emergency medical dispatch/ OR exp emergency medical dispatcher/ OR exp emergency medicine/ OR exp emergency patient/ OR exp emergency treatment/ OR exp emergency/ OR exp first aid/ OR exp intensive care/ OR exp paramedical personnel/ OR exp patient transport/ OR exp rescue personnel/ OR exp rescue work/ OR First aid.mp. OR First responder*.mp. OR Mobile Emergency Unit*.mp. OR Non conveyance.mp. OR Non-conveyance.mp. OR Out of hospital.mp. OR Paramedic*.mp. OR Patient Conveyance.mp. OR Patient transport*.mp. OR Patients transport*.mp. OR Pre Hospital.mp. OR Prehospital care.mp. OR Prehospital Emergency Care.mp. OR Prehospital.mp. OR Pre-hospital.mp. OR Rescue worker*.mp. OR Rescue.mp. OR Transportation of Patients.mp.) AND (Accreditation.mp. OR Adverse Event*.mp. OR Benchmark*.mp. OR Care standard*.mp. OR Clinical Audit.mp. OR Clinical Governance.mp. OR Donabedian model.mp. OR Efficacy.mp. OR Efficiency.mp. OR Evaluation.mp. OR exp accident prevention/ OR exp accreditation/ OR exp benchmarking/ OR exp clinical audit/ OR exp clinical competence/ OR exp clinical practice/ OR exp decision making/ OR exp health care quality/ OR exp health equity/ OR exp incident report/ OR exp job performance/ OR exp malpractice/ OR exp management/ OR exp medical audit/ OR exp medical error/ OR exp outcome assessment/ OR exp outcome assessment/ OR exp patient care/ OR exp patient safety/ OR exp patient satisfaction/ OR exp performance measurement system/ OR exp practice guideline/ OR exp productivity/ OR exp protocol compliance/ OR exp quality control procedures/ OR exp quality control/ OR exp risk management/ OR exp standard/ OR exp task performance/ OR exp time management/ OR exp time out/ OR exp time/ OR exp total quality management/ OR exp treatment outcome/ OR Guideline Adherence.mp. OR Health Care Quality, Access, and Evaluation.mp. OR Health Equity.mp. OR Incident Reporting.mp. OR Medical Error*.mp. OR Medical Error*.mp. OR Medical Mistake*.mp. OR "Outcome and Process Assessment (Health Care)".mp. OR "Outcome Assessment (Health Care)".mp. OR Outcome Assessment*.mp. OR Patient centered.mp. OR Patient Safety.mp. OR Patient Satisfaction.mp. OR Patient-Centered Care.mp. OR Performance indicator*.mp. OR Performance measure*.mp. OR "Process Assessment (Health Care)".mp. OR Process Assessment*.mp. OR Process Measure*.mp. OR Productivity.mp. OR Protocol Compliance.mp. OR Quality Assurance, Health Care.mp. OR Quality.mp. OR Risk Management.mp. OR Risks Management.mp. OR Standard of Care.mp. OR Task performance.mp. OR Time Management.mp. OR Time Out, Healthcare.mp. OR Time out.mp. OR Time plan.mp. OR Timeline.mp. OR Total Quality Management.mp. OR Utilisation.mp. OR Utilization.mp. OR Work Performance.mp.) AND (Abha.mp. OR Abu Dhabi.mp. OR Ahmadi.mp. OR Ajman.mp. OR Al Ain.mp. OR Al baha OR Al Batinah.mp. OR Al Buraimi.mp. OR Al Jawf.mp. OR Al Khor.mp. OR Al Madinah.mp. OR Al Wakrah.mp. OR Al Wusta.mp. OR Al-Ahsa.mp. OR Al-Qassim.mp. OR Arabian Gulf Countries.mp. OR Asir.mp. OR Bahrain.mp. OR Bahraini.mp. OR Budaiya.mp. OR Buraydah.mp. OR Dammam.mp. OR Dhofar.mp. OR Doha.mp. OR Dubai.mp. OR Emirati.mp. OR exp Bahrain/ OR Exp Kuwait/ OR Exp Oman/ OR Exp Qatar/ OR Exp Saudi Arabia/ OR Exp United Arab Emirates/ OR Fujairah.mp. OR Gizan.mp. OR Gulf Cooperation Council Countries.mp. OR Hafar Al-Batin.mp. OR Ha'il.mp. OR Hamad.mp. OR Hawalli.mp. OR Isa Town.mp. OR Jazan.mp. OR Jeddah.mp. OR Jizan.mp. OR Jubail.mp. OR Khamis Mushait.mp. OR Khobar.mp. OR Kuwait.mp. OR Kuwaiti.mp. OR Makkah.mp. OR Manama.mp. OR Mareb.mp. OR Mecca.mp. OR Medina.mp. OR Middle east/ OR Middle east.mp. OR Mubarak Al Kabeer.mp. OR Muharraq.mp. OR Musandam.mp. OR Muscat.mp. OR Najran.mp. OR Oman.mp. OR Omani.mp. OR Qasim.mp. OR Qatar.mp. OR Qatari.mp. OR Qatif.mp. OR Ras Al-Khaimah.mp. OR Riffa.mp. OR Riyadh.mp. OR Saudi Arabia.mp. OR Saudi.mp. OR Shabwah.mp. OR Sharjah.mp. OR Sitra.mp. OR Tabuk.mp. OR Ta'if.mp. OR UAE.mp. OR Umm Al Quwain.mp. OR United Arab Emirates.mp. OR Yanbu.mp.) | Result #  1694 |
